# Microbial potential for carbon and nutrient cycling in a geogenic supercritical carbon dioxide reservoir

**DOI:** 10.1111/1462-2920.13706

**Published:** 2017-05-02

**Authors:** Adam J.E. Freedman, BoonFei Tan, Janelle R. Thompson

**Affiliations:** ^1^ Department of Civil and Environmental Engineering Massachusetts Institute of Technology Cambridge MA USA; ^2^ Center for Environmental Sensing and Modeling Singapore‐MIT Alliance for Research and Technology Singapore Singapore

## Abstract

Microorganisms catalyze carbon cycling and biogeochemical reactions in the deep subsurface and thus may be expected to influence the fate of injected supercritical (sc) CO_2_ following geological carbon sequestration (GCS). We hypothesized that natural subsurface scCO_2_ reservoirs, which serve as analogs for the long‐term fate of sequestered scCO_2_, harbor a ‘deep carbonated biosphere’ with carbon cycling potential. We sampled subsurface fluids from scCO_2_‐water separators at a natural scCO_2_ reservoir at McElmo Dome, Colorado for analysis of 16S rRNA gene diversity and metagenome content. Sequence annotations indicated dominance of *Sulfurospirillum*, *Rhizobium, Desulfovibrio* and four members of the Clostridiales family. Genomes extracted from metagenomes using homology and compositional approaches revealed diverse mechanisms for growth and nutrient cycling, including pathways for CO_2_ and N_2_ fixation, anaerobic respiration, sulfur oxidation, fermentation and potential for metabolic syntrophy. Differences in biogeochemical potential between two production well communities were consistent with differences in fluid chemical profiles, suggesting a potential link between microbial activity and geochemistry. The existence of a microbial ecosystem associated with the McElmo Dome scCO_2_ reservoir indicates that potential impacts of the deep biosphere on CO_2_ fate and transport should be taken into consideration as a component of GCS planning and modelling.

## Introduction

Natural subsurface carbon dioxide reservoirs in the greater Colorado Plateau region serve as models for understanding the long‐term fate of CO_2_ after injection for geological carbon sequestration (GCS) (IPCC, 2007; Lal, 2008). While these reservoirs have been geologically characterized for commercial CO_2_ production (Stevens *et al*., 2001; Baines and Worden, 2004), the taxonomic and genomic diversity of microbial populations in these systems remain unknown. At reservoir depths typically >1 km, CO_2_ exists in the supercritical (sc) phase (≥31.1°C, ≥72.9 atm). Although scCO_2_ is regarded as a microbial sterilizing agent (White *et al*., 2006; Zhang *et al*., 2006; Mitchell *et al*., 2008; Ortuño *et al*., 2012), recent field and laboratory studies indicating resilience to stresses associated with supercritical (Mitchell *et al*., 2008; Mu *et al*., 2014; Peet *et al*., 2015) and near‐critical CO_2_ (de Beer *et al*., 2013; Emerson *et al*., 2015) suggest that scCO_2_‐bearing reservoirs may support a stable microbial biosphere.

Due to its non‐polar solvent chemistry, pure scCO_2_ may penetrate bacterial cell walls and membranes, extracting fatty acids, lipids and other intracellular materials from the cytosol (Ulmer *et al*., 2002). High concentrations of dissolved CO_2_ may decrease intracellular pH, disable enzymes, disrupt protein synthesis, and cause cellular desiccation, ultimately resulting in cell death (Spilimbergo and Bertucco, 2003; Zhang *et al*., 2006; Kirk, 2011). Natural emplacement, or injection of scCO_2_ during GCS, may also indirectly stimulate microbial growth by serving as a substrate for autotrophic metabolism, extracting nutrients from the subsurface organic matrix (Kharaka *et al*., 2006), and releasing redox substrates from dissolved minerals. Thus, exposure to scCO_2_ during natural or GCS processes may represent a major selective agent for microbial diversity.

The 800 km^2^ McElmo Dome scCO_2_ reservoir in southwestern Colorado (Supporting Information Fig. S1), operated by KinderMorgan, is the largest supplier of industrially produced CO_2_ in the world (Stevens *et al*., 2001). Estimates based on stable isotope data suggest CO_2_ began to accumulate 40 to 72 million years ago (Cappa and Rice, 1995; Gilfillan *et al*., 2008), a timescale during which assembly of scCO_2_‐tolerant microbial communities may have occurred. CO_2_ at McElmo Dome is trapped at depths of 1800 to 2600 m within the 100 m thick dolomite‐rich Leadville Formation (Allis *et al*., 2001; Gilfillan *et al*., 2009) where the CO_2_ exists as a supercritical fluid at an approximate temperature and pressure of 65°C and 135 atm respectively (Allis *et al*., 2001). Rich in permeable dolomites (CaMg(CO_3_)_2_), the Leadville Formation has an average porosity of 11%. The 400 m thick Paradox Salt Formation acts as a low‐permeability trapping layer above the Leadville Formation (Stevens *et al*., 2001). Wells penetrating the Leadville Formation produce biphasic gas‐brine mixtures from which CO_2_ is dehydrated and compressed, while the brine is re‐injected (Stevens *et al*., 2001). Fluid gas content includes 98.2% CO_2_, 1.6% N_2_, and 0.2% CH_4_ (Allis *et al*., 2001).

We hypothesized that the Leadville Formation would harbor a low biomass microbial ecosystem adapted to high pCO_2_ associated with geogenically emplaced scCO_2_ and the anoxic, low‐nutrient conditions typical of subsurface habitats (Phelps *et al*., 1994). To examine this, we sampled fluids from ten CO_2_ production wells and a pond containing well drilling fluids, comparing cell densities with element and nutrient profiles. Taxonomic and genomic diversity were characterized from two wells by analysis of 16S rRNA gene amplicons and microbial genomes resolved from metagenomes. Analysis of genomes with near complete coverage of single copy genes enabled prediction of metabolic capacity and syntrophy between microbial populations for biomass production, biogeochemistry and carbon cycling in the McElmo Dome scCO_2_ reservoir.

## Results

### Fluid geochemistry and suspended cell numbers

Onsite periodic testing of CO_2_ production wells by KinderMorgan revealed well temperatures of 59.8–78.1°C, and produced fluid ratios of 2021–5418 l H_2_O per liter of liquid CO_2_ based on measured CO_2_ gas volumes (R. Gersch, 2012; personal communication). ICP analysis of fluids obtained from ten wells (Supporting Information Table S3) indicated elemental concentrations between 0.32 and 16.7 g/l, consistent with on site salinity measurements (Table 1). Hierarchical clustering of normalized ICP signal intensities by Spearman rank correlation (Supporting Information Fig. S2) revealed two major sample clusters, where Cluster 1 corresponded to the most dilute samples with lowest H_2_O/CO_2_ ratios and enrichment in Mg, Fe, Ca, Al, Cr and Mn. Cluster 2 contains more concentrated samples with enrichment in Na, B, and As. Well location (i.e. Yellow Jacket field vs. Hovenweep field; Supporting Information Fig. S1) did not emerge as a significant driver of sample clustering (Supporting Information Fig. S2). The elemental composition of a sample from a nearby pond used as a source of well drilling fluid was most dilute (0.15 g/l) and clustered separately from well samples.

**Table 1:** Sample well test data and on site fluid measurement summary.

FIELD	WELL	NAME	WELL TEST DATA (KinderMorgan CO_2_)				MEASURED ON SITE	
			H_2_O (L * 10^3^)	CO_2_ (L * 10^3^)	(H_2_O/CO_2_) * 10^3^	Temp (°C)	pH^b^	Salinity (ppt)
Hovenweep	1	HA‐1	12.1	633.6	19.2	62.0	6.0	18
	2	HB‐5	16.4	1875.5	8.7	59.8	6.0	15
	3	HC‐2	3.5	1036.9	3.4	67.3	5.0	2
	4	HE‐1	1.4	675.3	2.0	68.2	5.0	1
	5	HF‐3	52.8	1391.6	37.9	74.5	6.0	15
Yellow Jacket	6	YA‐3	1.7	487.3	3.4	78.1	6.0	10
	7	YB‐4	42.9	791.2	54.2	74.8	6.0	25
	8	YC‐4	4.9	1883.1	2.6	66.0	5.5	2
	9	YD‐2	19.4	748.7	25.9	74.0	6.0	20
	10^a^	YF‐4	12.9	1530.2	8.4	75.3	6.0	10
–	Pond		–	–	–	27.0	5.5	0

**a.** Well 10 test results not available. CO2, H2O, and T data represent avg values for three nearby YF wells.

**b.** Following degassing, fluid pH values may be less acidic than in situ due to reduced carbonic acid content.

**Figure 1:**
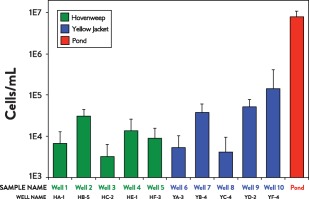
Cell concentrations/ml of ten McElmo Dome wells (five each from the Hovenweep and Yellow Jacket fields, respectively) and nearby pond that is a source for drilling fluid.

Epifluorescence microscopy of formaldehyde‐preserved samples revealed 3.2 × 10^3^ to 1.4 × 10^5^ cells/ml in produced fluid and 8.0x10^6^ cells/ml in drilling fluids pond water (Fig. 1). No trend in biomass density emerged by t‐test with respect to location (Hovenweep vs. Yellow Jacket; *p* = 0.20) or geochemical clustering (Cluster 1 vs. 2; *p* = 0.18).

### Taxonomic diversity in formation fluids and drilling fluids pond water

Nucleic acid extraction from two well samples (Wells 3 and 10) and the drilling fluids pond sample yielded PCR‐amplifiable DNA and were used as template for 16S rRNA gene amplicons targeting the V3‐V5 (clone libraries) and V3‐V4 (Illumina) hypervariable regions. No amplification was observed from no‐template controls or from genomic DNA from Wells 1‐2 and 4‐9 (attributed to PCR inhibition and/or insufficient template concentration). An archived false positive (AFP) from a discarded PCR run (i.e. a contaminated PCR negative control) was included to identify potential background laboratory contamination, which has recently been shown to be common and pervasive, especially in low biomass samples (Salter *et al*., 2014).

**Figure 2:**
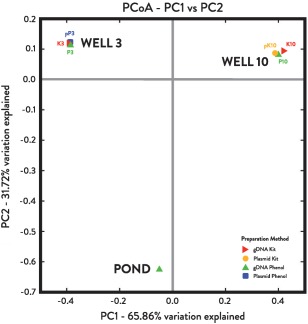
Beta diversity of 16S rRNA gene sequences from McElmo Dome well and drilling fluid pond. The first two axes of the UniFrac Principal Coordinate Analysis (PCoA) explain 97.58% cumulative percent variation. Samples cluster according to origin rather than DNA preparation method.

**Figure 3:**
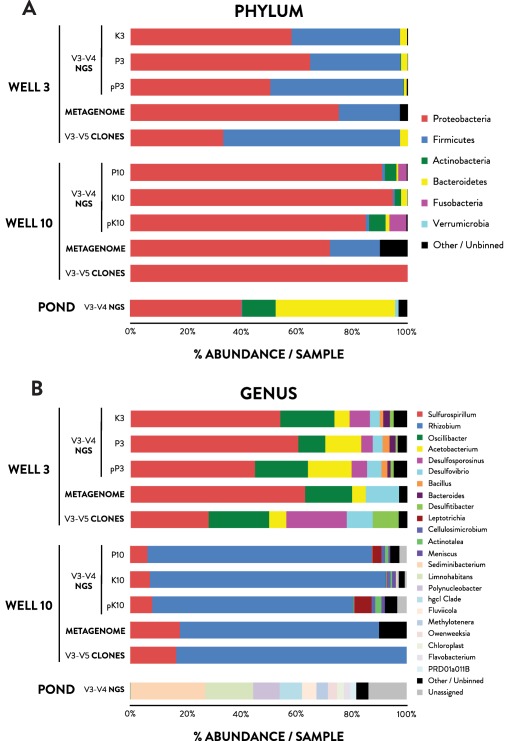
Taxonomic summary of the McElmo Dome microbial community constructed using RDP/Silva‐annotated 16S rRNA gene sequencing of clone and Illumina (NGS) libraries, and Illumina binned metagenome (MG) read frequencies on the (A) phylum and (B) genus level. K, P, pK, and pP refer to methods of DNA extraction and PCR template preparation, as described in experimental procedures (K = kit extracted gDNA, P = phenol extracted gDNA, p = clone library vector with 16S rRNA amplicon used as template).

Illumina V3‐V4 amplicon sequencing generated a total of 436 318 individual paired‐end reads from Well 3 and 10 samples, 96 626 from the pond, and 103 714 from the AFP. Following OTU clustering, comparison to the AFP resulted in the removal of 5.2–23.5% of reads per library (Supporting Information Tables S2 and S5). The most highly represented genera in the AFP (*Cloacibacterium*, *Acidovorax*, *Brevundimonas* and *Halomonas*) are considered to be reagent or laboratory contaminants. After decontamination, sequences from the two wells (Well 3 = 130 824; Well 10 = 62 334) formed 290 total OTUs, with Well 3 and Well 10 clustering into 187 and 199 OTUs respectively, while the pond (39 844 reads) clustered into 234 OTUs (Supporting Information Table S2). All clone library genus annotations were represented in Illumina OTUs (Fig. 3B; Supporting Information Table S4). PCoA analysis of UniFrac distances (Fig. 2) and rarefaction analysis (Supporting Information Fig. S3) indicated that diversity in well and pond samples was recovered to near‐completion and reflected distinct microbial communities unique to each sample and independent of DNA preparation method (Figs 2 and 3).

The majority of Illumina OTUs were assigned taxa by SINA/RDP (99.5% to phylum and 68.7% to genus). V3‐V5 clone libraries (54 sequences, 692 bp average length; Supporting Information Table S4) enabled species‐level taxonomic resolution for corresponding Illumina OTUs. 11 and 13 Bacterial phyla were recovered from Well 3 and 10 respectively (Fig. 3A; Supporting Information Fig. S4). No Archaeal phyla were recovered despite use of universal PCR primers. Most sequences from Well 3 (W3) and 10 (W10) samples were classified as Proteobacteria (W3: 57.7%, W10: 90.1%), which were dominated by OTUs corresponding to *Sulfurospirillum deleyanium* (W3: 31.8%, W10: 7.3%) and *Sulfurospirillum multivorans* (W3: 20.8%, W10: 0.1%), two OTUs corresponding to *Rhizobium petrolearium* in Well 10 (65.1%; 14.1%), and one OTU from *Desulfovibrio marrakechensis* in Well 3 (4.1%) (Fig. 3B and Supporting Information Fig. S4; Table 2). Both the *Sulfurospirillum* and *Rhizobium* sequences share the highest nucleotide identity with strains previously detected in oil fields or subsurface environments (Zhang *et al*., 2012; Tan and Foght, 2014). Firmicutes sequences recovered in high abundance in Well 3 (39.9%) correspond to several Clostridiales OTUs: *Oscillibacter valericigenes* (17.0%), *Acetobacterium carbinolicum* (10.8%), *Desulfosporosinus orientis* (6.0%) and *Desulfitibacter* (1.1%). In contrast, Firmicutes OTUs were in low abundance in Well 10 (1.0%), as were those affiliated with Actinobacteria (4.1%) and Fusobacteria (3.2%). Diversity in the drilling fluids pond was dominated by genera typical of freshwater surface environments (e.g. *Sediminibacterium*, *Limnohabitans*, *Polynucleobacter*, *hgcI_clade*, *Fluviicola*; Fig. 3B) and enriched in Bacteroidetes and Actinobacteria. The highly distinct pond community (Figs 2 and 3) suggests that drilling fluids are not a major source of microbial diversity in recovered formation fluids.

**Table 2:** Overview of genomes detected in the metagenome.

Well Sample	N50	Taxonomic affiliation based on 16S rRNA and single copy gene assignment	Binned genome size, Mbp (contig size range, bp) [reference genome size]^a^	Blastn 16S rRNA taxonomic affiliation of against NCBI NR database (full length unless stated)	Blastn comparison of binned 16S rRNA gene to Illumina generated OTU	# Contigs	# cds	Taxonomic distribution of ORF	Genome Completeness^b^	Abundance in Metagenome (%)	Abundance of Corresponding OTU (%)
10	36 674	*Sulfurospirillum* M102	2.6 (1000–24 067) [2.3–3.2]	*Sulfurospirillum deleyianum* DSM 6946 (98%)	OTU 6 (100%)	590	2559	Campylobacterales (92%); Epsilonproteobacteria (95%)	100%	18	7
		*Rhizobium* MD101	6.8 (1015–292 738) [4.9–7.5]	*Rhizobium petrolearium* strain SL‐1 (100%)	OTU 2 (100%)	164	6755	Rhizobiales (88%); Alphaproteobacteria (93%)	100%	72	65
3	14 942	*Desulfovibrio* MD33	5.0 (1001–68 886) [3.2–5.25]	*Desulfovibrio marrakechensis* strain EMSSDQ4 (98%)	OTU 47 (99.2%)	767	4545	Desulfovibrionales (92%)	100%	12	4
		*Acetobacterium* MD34	4.0 (1011–114 001) [4.04–4.05]	*Acetobacterium carbinolicum* (100%)	OTU 21 (100%)	315	3847	Eubacteriaceae (73%); Clostridiales (86%); Firmicutes (94%)	100%	5	11
		*Sulfurospirillum* M31^c^	2.6 N/A [2.3–3.2]	*Sulfurospirillum deleyianum* DSM 6946 (97%) Sulfurospirillum sp. M10 (100%); 523 bp fragment	Binned fragment does not overlap with Illumina OTU; OTU 6^e^	34	2776	Campylobacterales (92%); Epsilonproteobacteria (95%)	100%	36	32
		*Sulfurospirillum* M32	3.2 N/A [2.3–3.2]	*Sulfurospirillum* sp. JPD‐1 (99%); 676 bp fragment	Binned fragment does not overlap with Illumina OTU; OTU 626^e^	29	3095	Campylobacterales (88%); Epsilonproteobacteria (92%)	100%	27	21
		*Oscillibacter* MD34	3.2 (1013–160 132) [3.1–4.4]	*Oscillibacter valericigenes* strain Sjm18‐20 (96%)	OTU 12 (100%)	130	3278	Oscillospiraceae (42%); Clostridiales (66%); Firmicutes (88%)	100%	17	17
		*Desulfosporosinus sp*.	Not binned	Peptococcaceae *Desulfosporosinus orientis* (OTU affiliation)	No fragment available; OTU 44^a^	N/A		Peptococcaceae (100%)	N/A		6

^a^Reference genome sizes obtained from related genera at the NCBI genome database.

^b^Genome completeness computed based on comparison to 108 single copy genes (SGC) detected in bacterial genomes.

^c^Method for binning two Sulfurospirillum strains in Well 3 discussed in Supporting Information.

### Diversity of genomes recovered from metagenome sequences

**Figure 4:**
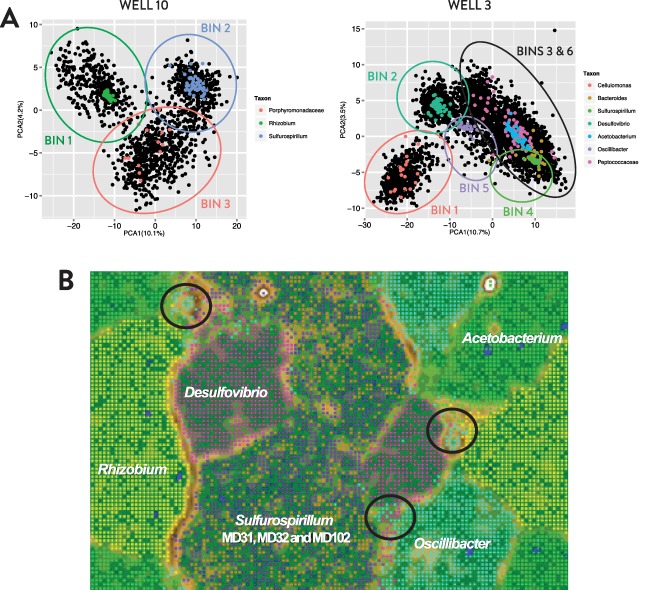
A. Principal component analyses of the tetranucleotide frequencies of metagenomes from Wells 3 and 10. Each black dot represents a single contig/scaffold of 1–500 kbp. Contigs containing single copy gene(s) are overlaid with dots where colors represent different bacterial taxa. In circles are contigs (crude bin) extracted for further decontamination based on both homology and compositional‐based approaches, after which contigs in crude bins belonging to unexpected taxa were placed in their correct bin. B. Emergent Self Organizing Map (ESOM) of the tetranucleotide frequencies of contigs in binned genomes. Each dot represents a 2000 bp scaffold/contig fragment. Dots are colored according to genomic bins presented in Table 2. The region labelled as *Sulfurospirillum* contains two and one strain of *Sulfurospirillum* detected in Metagenome 3 and 10 respectively. In areas where dots with different colors appeared to be ‘mixed’ (circles in plot), the entire contig (ORFs) of each fragment was examined for their consistency in sequence homology (Blastp against NCBI NR‐database). In all cases, these fragments were part of a long contig (of which other fragments were located in the correct region of the ESOM map). In some cases, fragments that are ‘mixed’ were related to mobile genetic elements, which may have resulted in differences in GC content and therefore tetranucleotide frequencies. The three strains of *Sulfurospirillum* were subsequently separated using homology‐based approaches (supplementary methods).

A total of 18.7 Gb of metagenomic sequence was generated from McElmo Dome samples. Metagenome assemblies from Wells 3 and 10 had an N50 of 14 942 and 36 674 bp with an average coverage of 20.7X and 35.4X respectively (Supporting Information Table S2). Reconstructed genomes using homology and compositional‐based approaches (Figs 4A and B) yielded six and two near‐complete genomes with >99% single copy genes (SCG) detected from Wells 3 and 10 respectively (Table 2). Binned genome sequences represent the vast majority of total reads (97% and 90% for Wells 3 and 10 respectively). For the remaining ∼3% of metagenomic reads obtained from Well 3, contigs were assigned to predicted partial genomes from *Desulfosporosinus* (95% SCG), *Bacteroides* (30% SCG) and *Cellulomonas* (24% SCG). Partial genomes were unable to be binned due to insufficient contig length (<2kb), low coverage (<20X; Supporting Information Fig. S5) and clustering with other Firmicutes‐affiliated contigs (Supporting Information Fig. S5). The detection of a *Desulfosporosinus* population in the metagenome was supported by phylogenetic placement of genes recovered from unbinned sequences (Supporting Information Fig. S7, *dsr*AB; Supporting Information Fig. S8, *bss*A) and Illumina OTU annotation. The ∼10% of Well 10 reads that were not associated with *Rhizobium* and *Sulfurospirillum* were assigned to Porphyromonadaceae (19% SCG) and other unknown microbes (<5% SCG). Our Emergent Self Organizing Map (ESOM) showed clear separation of contigs between genomic bins (Fig. 4B). The taxonomic affiliation and sequence distribution of binned genomes were consistent with abundance profiles of Illumina 16S rRNA gene OTUs and cloned 16S rRNA genes (Table 2; Fig. 3). The apparent increase in abundance of ‘Other/Unbinned’ sequences in Well 10 relative to Well 3 based on metagenomic analysis (Fig. 3) was due to the detection of fewer taxonomically‐informative genes and a higher percentage of contigs with conflicting taxonomic assignments rather than the identification of novel species.

### Functional capacity of microbial genomes

Sequences recruited to the three *Sulfurospirillum* binned genomes represented the most and second most reads in the Well 3 (63%) and Well 10 (18%) metagenomes respectively. *Sulfurospirillum* genomes MD31 and MD102 were most closely related to *S. deleyanium* (98% 16S ID), while MD32 more closely resembled *S. multivorans* (98% 16S ID) (Table 2 and Supporting Information Table S4). All *Sulfurospirillum* genomes annotated using RAST and IMG harbor predicted genes for chemotactic motility (*che* and *fla* genes), nitrogen fixation (*nif*DKH) and access to multiple electron acceptors for respiration including nitrate (*nap*AB), arsenate (*arr*AB) and fumarate (fumarate reductase) (Table 3), consistent with activities observed in closely related strains (Magnuson *et al*., 1998; John *et al*., 2009; Goris *et al*., 2014). *Sulfurospirillum* genomes predict organic carbon transporters for lactate, formate, gluconate, peptides and amino acids, indicating potential uptake for heterotrophic carbon metabolism via glycolysis, the TCA cycle, and several fermentation pathways (Table 3 and Supporting Information Table S6C). Mixotrophic growth fueled by inorganic electron donors hydrogen and sulfur is documented in cultured strains of *Sulfurospirillum* (Finster *et al*., 1997) and is indicated in the three McElmo Dome *Sulfurospirillum* genomes (MD31, MD32 and MD102) by annotation of *sox*ABXYZ, *sor*AB and *yed*Y genes for the sulfur oxidation pathway, (which can be coupled to nitrate reduction for anaerobic energy conservation (Eisenmann *et al*., 1995)), and biosynthetic pathways for hydrogenases which catalyze hydrogen oxidation (i.e. *hyp*ABCDEF and *hyf*ABCEF for biosynthesis of NiFe hydrogenase and Hydrogenase 4 respectively).

**Table 3:** Metabolic potential of binned genomes in Wells 3 and 10.

Pathway/function	Key enzymes	Well 3						Well 10	
		Ac34	Os35	Dv33	Ss31	Ss32	Ds*	Rz101	Ss102
**Sulfur metabolism**									
**Dissim. Sulfate Reduction**	*sat/cysND, aprAB*								
**Dissim. Sulfite Reduction**	*dsrAB*	R							
**Sulfur Oxidation**	*soxABXYZ*								
**Sulfite Oxidation**	*sorAB, yedY*								
**Nitrogen metabolism**									
**Dissim. Nitrate Neduction**	*narGHIJ, napAB*	R		I					R
**Dissim. Nitrite Reduction**	*nirBD, nrfADH*							I	
**Denitrification**	*nirSK, norBC, nosZ*								
**Anammox**	*hzo*								
**Nitrogen Fixation**	*nifDKH, anfG*								
**Metals/metalloids metabolism**									
**Fe(III) Reduction (respiration)**	*frd*	I							
**Fe(II) Oxidation**	*qcr*		R	R					
**As(V) Reduction (respiration)**	*arrAB*				R				R
**As(V) Reduction (detox)**	*arsC*								
**As(III) Oxidation (assimlation)**	*aoxAB*		I		I	I			
**Organic electron acceptors**									
**Fumarate ‐> Succinate**	*Fumarate reductase*								
**CO_**2**_ Fixation**									
**Wood‐Ljungdahl**	*CODH/acsAB*								
**Reverse TCA Cycle**	*aclBA, oorDABC, porCDAG/nifJ*								
**Calvin Cycle**	*Rubisco*	I	I	I					
**Carbonic Anhydrase**	*CA*								
**Phosphoenolpyruvate carboxylase**	ppc								
**Carbamoyl‐phosphate synthase**	carAB								R
**Fermentation**									
**Acetyl‐CoA ‐> Acetate**	*Acetate kinase*			R				I	
**Acetyl‐CoA ‐> Butyrate**	*Butyrate kinase*							R	
**Pyruvate ‐> Formate**	*Pyruvate‐formate lyase*				I				I
**Pyrvuate ‐> Lactate**	*Lactate dehydrogenase*								
**Formate ‐> H_**2**_ + CO_**2**_**	*Formate‐hydrogen lyase*							R	
**Acetaldehyde ‐> Ethanol**	*Aldehyde dehydrogenase*				I			I	I
**Butyraldehyde > Butanol**	*Butanol dehydrogenase*				R	R			R
**Acetolactate ‐> 2,3‐Butanediol**	*Acetolactate decarboxylase*								
**Mixed Acid General**	*Alcohol dehydrogenase*			I		I		I	
**Central carbon metabolism**									
**Glycolysis/E‐D**	*FBPase; G6P‐Iso; PGDH*								
**TCA Cycle**	*Citrate synthase; Isocitrate DH*	I	I						
**Pentose Phosphate**	*Transketolase; Rib‐5‐P isomerase*				I	I			
**Anaerobic hydrocarbon activation**									
**Benzene**	*abcD* *								
**Alkane**	*assA* *								
**Toluene**	*bssA* *								
**Ethylbenzene**	*ebdA* *								
**Tyrosol**	*HPAH*								
**Hydrogenase**									
**Hydrogenase 1**	*Hya*			R					
**Hydrogenase 2**	*Hyb*			I	I	I			I
**Hydrogenase 3**	*Hyc*			R	I				I
**Hydrogenase 4**	*Hyf*								
**Fe Hydrogenase**	*Hyd*	R	R						R
**FeFe Hydrogenase**	*Hym*								
**NiFe Hydrogenase Biosynthesis**	*Hyp*				I				
**Energy Conserving Hydrogenase**	*Ech*			I					
**NADP‐Reducing Fe hydrogenase**	*Hnd*	I		I					
**NAD‐Reducing hydrogenase**	*Hox*	I	R						
**Membrane bound hydrogenase**	*Mbh*								

LEGEND							
Well	Bin	Strain	Color	Annotation	Label		Annotation
**3**	**Ac34**	*Acetobacterium* MD34		All required enzymes			**BOTH** RAST & IMG‐KO
	**Os35**	*Oscillibacter* MD35		Associated enzymes	**R**	**R**	**RAST** ONLY
	**Dv33**	*Desulfovibrio* MD33		No associated enzymes	**I**	**I**	**IMG** ONLY
	**Ss31**	*Sulfurospirillum* MD31		Annotation unavailable			
	**Ss32**	*Sulfurospirillum* MD32					
	Ds*	*Desulfosporosinus*					
**10**	**Rz101**	*Rhizobium* MD101					
	**Ss102**	*Sulfurospirillum* MD102					

*Desulfosporosinus (Ds) genes were assigned function based on GC content and sequence coverage using HMM based on best Blastp hit. When a key gene or subunit is not detected in a genomic bin based on RAST annotation, tBlastn and HMM were used to screen the entire metagenome, followed by Blastp searches (for taxonomic affiliation) against the NCBI database to verify the absence of such function in the metagenome.

The ability to couple oxidation of inorganic electron donors, such as hydrogen and sulfur, to CO_2_ fixation for chemolithoautotrophic growth has not been demonstrated within the genus *Sulfurospirillum* (Kelly and Wood, 2006). However, a partially annotated Wood‐Ljungdahl pathway within the genome of *Sulfurospirillum* MD32 suggests the potential capacity for redox coupled CO_2_ utilization. The Wood‐Ljungdahl pathway requires two enzymes, carbon monoxide dehydrogenase (CODH) (AcsA), and an acetyl‐CoA synthase (AcsB). Additional enzymes in the pathway include CooC (CODH nickel insertion), acetyl‐CoA synthase iron‐sulfur proteins, and several CODH subunits. An *acs*B gene was detected in the MD32 genome adjacent to several key pathway components, including accessory protein, CooC (on RAST contig 10/IMG contig 128), as well as CODH‐associated genes *coo*A, *coo*L, *coo*X, *coo*U, *coo*H and *coo*F on a single contig (Rast contig 6/IMG contig 125). However, the gene for carbon monoxide dehydrogenase (*coo*S/*acs*A) was not identified despite its previous detection in other *Sulfurospirillum* strains (Jensen and Finster, 2005; Tan and Foght, 2014). Therefore, while the presence of *acs*B has conventionally been used as a marker for CO_2_ fixation and acetogenesis (Gagen *et al*., 2010), the absence of *acs*A suggests that a partially or alternatively functional Wood‐Ljungdahl pathway is present in *Sulfurospirillum* MD32. All *Sulfurospirillum* genomes contain genes encoding carbonic anhydrase, which facilitates CO_2_ conversion to bicarbonate (Smith and Ferry, 2000), and the bicarbonate incorporating enzyme carbamoyl‐phosphate synthase for pyrimidine and arginine biosynthesis (Arioli *et al*., 2009). Taken together, the McElmo Dome *Sulfurospirillum* genomes are predicted to be capable of growth under conditions of low nitrogen, using bicarbonate, potentially CO_2_, and fermentative substrates as carbon and/or energy sources, with access to diverse substrates for anaerobic respiration including arsenate and nitrate, both of which are detected in formation fluids.

Metagenome 3 yielded three additional putatively complete genomic bins (*Acetobacterium* MD34, *Desulfovibrio* MD33 and *Oscillibacter* MD35). Notably, these genomes also contain RAST and IMG‐predicted genes for N_2_ fixation (*nif*DHK), chemotactic motility (*che* and *fla*), carbonic anhydrase and bicarbonate‐utilizing enzymes such as carbamoyl‐phosphate synthase (all genomes) and phosphoenolpyruvate carboxylase (MD34 only), which allows entry of inorganic carbon into central metabolism (Table 3 and Supporting Information Table S6B). In addition, all genomes harbored predicted transporters or pathways for amino acid and peptide uptake, suggesting the ability to recycle organic materials released by cell lysis or exudation (Supporting Information Table S6C).

Chemolithoautotrophy within the Well 3 community is supported by annotation of a complete Wood‐Ljungdahl CO_2_‐fixation pathway and pathways for biosynthesis of FeFe and NiFe hydrogenases for H_2_ oxidation in the *Acetobacterium* MD34 genome (Tables 3 and Supporting Information Table S6B), consistent with the growth physiology of close relative, homoacetogen *A. carbinolicum* (100% 16S rRNA gene identity) (Eichler and Schink, 1984). In addition, annotation of a phosphoenolpyruvate carboxylase gene, which catalyzes the irreversible addition of bicarbonate to phosphoenolpyruvate and is hypothesized to promote tolerance of high pCO_2_ conditions (Arioli *et al*., 2009; Santillan *et al*., 2015), indicates a possible entry point for inorganic carbon to central metabolism. *Acetobacterium* MD34 may be capable of mixotrophy as indicated by annotation of transporters and permeases for uptake of lactate, sugar monomers and ethanol with energy conservation by mixed acid fermentation (Table 3). In addition, annotation of a respiratory sulfite reduction pathway and a non‐energy generating As(V) reduction pathway associated with detoxification may represent adaptations to high As and S levels in formation fluids (Supporting Information Table S3).

The genome of *Desulfovibrio* MD33 is most closely related to the sulfate reducing bacteria *D. marrakechensis* strain EMSSDQ4 (98% 16S rRNA gene identity) and contains a complete pathway for respiration by sulfate (*sat*/*dsr*AB) and nitrite reduction (*nrf*AH) as well as an incomplete denitrification pathway (*nir*S, *nor*B; Tables 3 and Supporting Information Table S6A). Heterotrophic potential is indicated by annotation of transporters for lactate, glycerol, fructose and other sugar monomers (Supporting Information Table S6C), which may be processed via central carbon metabolism or several fermentative pathways (Table 3). MD33 mixotrophic growth potential is further supported by predicted access to inorganic electron donors Fe(II) (Fe(II)‐cytochrome c reductase) and molecular hydrogen (FeFe, NiFe hydrogenases) as previously demonstrated for cultured *Desulfovibrio* strains (Romão *et al*., 1997; Roseboom *et al*., 2006; Timóteo *et al*., 2012). A partially annotated Wood‐Ljungdahl pathway (CODH catalytic subunit CooS/AcsA clustered with accessory proteins CooF, CooC and CooA on Rast contigs 75/819 and IMG contig 128) may indicate additional capacity for C1 metabolism.

Annotations from the Well 3 binned genome of *Oscillibacter* MD35 (closest relative *O. valericigenes* Sjm18‐20 with 96% 16S rRNA gene identity; Katano *et al*., 2012) are indicative of a heterotroph able to uptake and metabolize lactate, glycerol, glutamate and several sugars. Energy conservation may occur by fermentation, anaerobic respiration by utilizing As(V) as an electron acceptor or through incomplete denitrification (Table 3 and Supporting Information Table S6A). Predicted fermentation end products formate, lactate and ethanol (Table 3) may contribute to an anaerobic food web. Fe(II)‐cytochrome c reductase annotation suggests *Oscillibacter* MD35 may also access Fe(II) as an electron donor for mixotrophic growth. However, a full suite of predicted sporulation genes suggests the possibility the MD35 is metabolic dormant *in situ*.

The presence of a sulfate‐reducing *Desulfosporosinus* population in Well 3 is supported by 16S rRNA gene data and detection of genes phylogenetically affiliated with *Desulfosporosinus* spp., including dissimilatory sulfite reductase genes *dsr*AB (Supporting Information Fig. S7) and the *bss*A gene encoding the alpha‐subunit of benzylsuccinate synthase (Supporting Information Fig. S8). The BssA enzyme is involved in the anaerobic degradation of monoaromatic hydrocarbons via fumarate addition, and *Desulfosporosinus* has been routinely implicated in subsurface degradation of toluene coupled to sulfate reduction (Liu *et al*., 2004; Lee *et al*., 2009). As McElmo Dome overlies minor hydrocarbon deposits (Rabinowitz and Janowiak, 2005) it is possible that these reduced compounds represent accessible sources of carbon and energy.

The Well 10 metagenome was dominated (72% of sequences) by the *Rhizobium* MD101 genome, which displays 100% 16S rRNA gene identity to an oil‐contaminated soil isolate, *Rhizobium petrolearium* strain SL‐1. Annotation of a sulfur oxidation pathway (soxABXYZ) and complete Calvin Cycle indicates the potential for autotrophic growth and chemosynthesis, as has been previously described for *Rhizobium* isolates from calcareous desert soils (El‐Tarabily *et al.*, 2006). The RuBisCO gene detected in the *Rhizobium* MD101 genome phylogenetically clusters with type IC and ID forms (Supporting Information Fig. S9), which are typically associated with mixotrophs, including members of the Rhizobiales order (Badger and Bek, 2007). Carbon monoxide dehydrogenase (AcsA) and carbonic anhydrase may catalyze utilization of CO_2_ and other C1 compounds including carbon monoxide as an electron donor (*cox*LMS; Cunliffe, 2011). Annotated fermentative pathways and transporters for formate, glucose, xylose, fructose, lactose, peptides and amino acids (Supporting Information Table S6C) suggest that MD101 may not rely on obligate autotrophy for growth. Heterotrophic or autotrophic metabolism may be coupled to a full denitrification pathway (*nir*K, *nor*BC, *nos*Z; Table 3) or anaerobic respiration, utilizing iron, nitrate (*nap*AB), nitrite (*nir*BD) or fumarate as electron acceptors (Supporting Information Table S6A). The MD101 genome predicts chemotactic motility (*che* and *fla* genes), but notably lacks genes for nitrogen fixation capacity ubiquitous among other McElmo Dome genomes.

## Discussion

The first insights into the taxonomic and genomic diversity within a natural scCO_2_ reservoir biosphere have been revealed through 16S rRNA gene and metagenome sequencing analysis. Strong agreement in distribution of the most highly represented taxonomic groups between 16S rRNA gene amplicons and metagenomic sequences (Fig. 3; Table 2) suggests a limited effect of biases associated with PCR amplification. Based on both sequencing approaches, the microbial assemblages in formation fluids appear to be dominated by two microbial taxa from Well 10 (*Rhizobium petrolearium* MD101 and *Sulfurospirillum deleyianum* MD102) and six taxa from Well 3 (*Sulfurospirillum deleyianum* MD31*, Sulfurospirillum multivorans* MD32, *Desulfovibrio marrakechensis* MD33*, Acetobacterium carbinolicum* MD34*, Oscillibacter valericigenes* MD35 and *Desulfosporosinus orientis*). The abundance of these genera (and lower abundance taxa, e.g. those within taxonomic classes Bacteroidia, Bacilli, Fusobacteria, Actinobacteria) and their previous detection in deep subsurface anoxic environments (Suess *et al*., 2005; Rastogi *et al*., 2010; Itävaara *et al*., 2011; Marshall *et al*., 2013; Engelhardt *et al*., 2013; LaBelle *et al*., 2014) suggest the presence of a microbial biosphere in scCO_2_‐exposed formation fluids. Overall, the richness and diversity of 16S rRNA gene sequences identified in Wells 3 and 10 (290 OTUs) is similar to observations from other deep subsurface and/or high pCO_2_ systems (e.g. 311 OTUs in fluids from a high pCO_2_ near‐surface geyser (Emerson *et al*., 2015); 176‐435 OTUs in post‐scCO_2_ injection formation fluids collected from 1400 m depth (Mu *et al*., 2014); 160‐340 OTUs from deep subsurface formation fluids (Chehoud & Onstott, 2011; Wuchter *et al*., 2013)). The shared presence and similar proportions of high and low abundance microbial taxa between McElmo Dome and other subsurface habitats may suggest similar drivers for subsurface diversity (e.g. nutrient scarcity).

Metabolic annotations of recovered genomes using the RAST and IMG functional databases predict a potential food web based on remineralization of organic carbon or primary production via chemolithoautotrophy where inorganic electron donors may include reduced sulfur (*sox*ABXYZ genes in *Rhizobium* MD101 and *Sulfurospirillum* MD31, MD32 and MD102), hydrogen (FeFe and NiFe hydrogenases in *Desulfovibrio* MD33, NiFe hydrogenase in all *Sulfurospirillum* strains) or iron (Fe(II)‐cytochrome c reductase in *Desulfovibrio* MD33 and *Oscillibacter* MD35). Annotation of pathways for anaerobic respiration using diverse electron acceptors (e.g. nitrate, sulfate, As(V)) or fermentation indicates mechanisms for energy conservation. Challenges associated with low dissolved nitrogen appear offset by the near ubiquitous capacity for nitrogen fixation where *nif*DHK genes encoding the nitrogenase complex are detected in all genomes except *Rhizobium* MD101.

**Figure 5:**
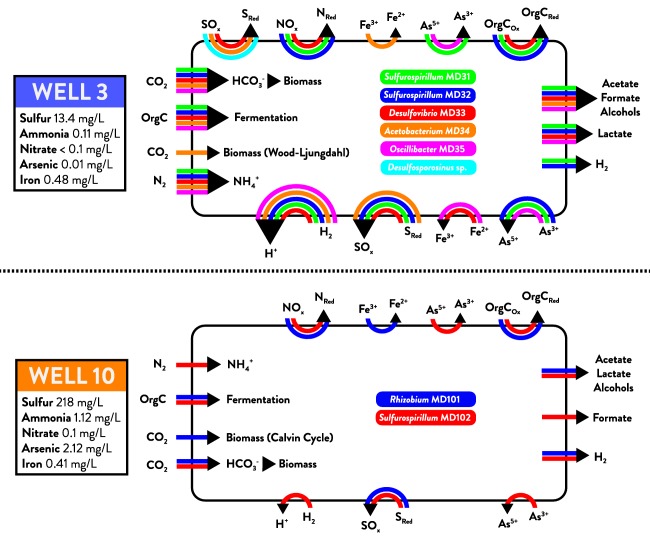
Model of potential interactions and dependencies among populations in the microbial assemblages recovered from Well 3 and Well 10. The microbial community metagenome is represented by large black rectangles (Well 3 above, Well 10 below). Within each metagenome, genome bins are assigned to colors and predicted metabolic functions for each genome are represented by colored arrows between reactants and products. Predicted functions indicated at the top and bottom edges of the diagram represent reduction and oxidation reactions respectively. Arrows on the left edge include carbon and nitrogen fixation potential and predicted capacity for fermentative metabolism. Arrows on the right edge represent predicted fermentative byproducts that would be generated and potentially available for uptake by community members via an anaerobic food web. Measured fluid elemental concentrations for both wells are listed at left.

Ecosystem interaction among McElmo Dome populations is suggested by metabolic potential and recovery of similar microbial assemblages around the world. The Well 3 assemblage resembles a chemolithoautotrophic ecosystem in anoxic subglacial volcanic‐fed lakes in Iceland that is comprised of *Sulfurospirillum, Acetobacterium* and *Desulfosporosinus* (Gaidos *et al*., 2009). A similar community of *Sulfurospirillum, Acetobacterium* and *Desulfovibrio* was enriched from brewery wastewater as members of an electrosynthetic microbiome capable of CO_2_ fixation and H_2_ or acetate production using electrodes as an electron donor (Marshall *et al*., 2013; LaBelle *et al*., 2014). In both cases, *Acetobacterium* is proposed to play a major role in fixing CO_2_ into compounds including formate or acetate that can be accessed by other populations engaged in sulfur cycling (sulfur oxidation and sulfate reduction). Competition between sulfate‐reducing *Desulfovibrio* and nitrate‐reducing *Sulfurospirillum* spp. for organic electron donors has been shown to reduce rates of sulfide production and associated souring in oil fields (Hubert and Voordouw, 2007). Similarly, the two taxa that make up the majority of the Well 10 assemblage, *Rhizobium* and *Sulfurospirillum*, are ubiquitous in petroleum reservoir formation waters (Zhang *et al*., 2012; Gao *et al*., 2016) and are also widely associated with high arsenic environments (Lloyd and Oremland, 2006; Chang *et al*., 2010). The Well 10 assemblage may also represent a chemolithoautotrophic ecosystem based on CO_2_ fixation fueled by sulfur oxidation by *Rhizobium* MD101 and carbon remineralization by *Sulfurospirillum* MD102. A schematic overview of ecosystem metabolism predicted by Well 3 and 10 genomes is presented in Fig. 5.

We hypothesized that abundantly available CO_2_ at McElmo Dome would select for autotrophic and CO_2_‐utilizing bacteria. Thermodynamic models also suggest the favorability of CO_2_‐consuming metabolic reactions (e.g. acetogenesis) would increase in scCO_2_‐exposed systems relative to atmospheric conditions (Onstott, 2005; Kirk, 2011; West *et al*., 2011). Support for this hypothesis is found in binned genomes from both wells, which revealed complete CO_2_ fixation pathways in Well 3 *Acetobacterium* MD34 (Wood‐Ljungdahl Pathway) and Well 10 *Rhizobium* MD101 (Calvin Cycle). Though speculative, several incomplete Wood‐Ljungdahl pathways (Table 3 and Supporting Information Table S6B) that include annotation of CO dehydrogenase (*acs*A) in *Desulfovibrio* MD33 and acetyl‐CoA synthase (*acs*B) in *Sulfurospirillum* MD32 may reflect limited CO_2_ fixation capacity or alternative metabolic functions. For example, *Dehalococcoides mccartyi* leveraged an incomplete Wood‐Ljungdahl pathway for one‐carbon metabolism coupled to amino acid biosynthesis (Zhuang *et al.*, 2014) while Nakayama *et al*. (2014) argued that endosymbiont *Epithemia turgida* utilizes a partial Calvin Cycle (absent RuBisCO) to catabolize exogenously supplied carbohydrates. Notably, metagenome sequences from the Crystal Geyser system also contained CO_2_‐fixation signatures via the Calvin Cycle (Emerson *et al*., 2015) and Wood‐Ljungdahl Pathway (Probst *et al*., 2016), suggesting similar modes of carbon cycling may be employed across high pCO_2_ systems. In addition to autotrophic fixation, CO_2_ can be incorporated into cell biomass through anaplerotic reactions. For example, the marine bacteria SAR11 can obtain up to 30% of its cellular carbon through this mechanism (Palovaara *et al*., 2014). All McElmo Dome genomes encode predicted enzymes for anaplerotic incorporation of CO_2_ including conversion of CO_2_ to H_2_CO_3_ (carbonic anhydrase) and incorporation of bicarbonate for biomass building (carbamoyl‐phosphate synthase, phosphoenolpyruvate carboxylase), suggesting CO_2_ may be widely accessed as a substrate during growth.

Sulfur is dissolved in fluids from both Well 3 (13.4 mg/l) and Well 10 (218 mg/l), although *in situ* oxidation states are not known based on ICP results. Genomes from *Sulfurospirillum* MD31, MD32, MD102 and *Rhizobium* MD101 carry the full suite of *sox* genes for sulfur or sulfide oxidation while sulfite has the potential to be oxidized via sulfite oxidase/hydrogenase (Table 3) by all taxa except *Oscillibacter* MD35. Because Well 3 populations (i.e. *Desulfovibrio* MD33, *Acetobacterium* MD34, *Desulfosporosinus*) may access oxidized sulfur compounds as electron acceptors for dissimilatory sulfate and sulfite reduction, the order of magnitude lower sulfur concentration in Well 3 relative to Well 10 may be due to loss of H_2_S during fluid degassing or sulfide mineral precipitation. We therefore hypothesize that sulfur‐bearing redox substrates provide energy to fuel the persistence of the carbonated biosphere, and may also strongly impact local geochemical conditions.

The nitrogen cycle is accessible to microbial communities at McElmo Dome as N_2_ gas (1.6% total gas), nitrate (Well 3: b.d.; Well 10: 0.10 mg/l) and ammonium (Well 3: 0.11 mg/l; Well 10: 1.12 mg/l) (Supporting Information Table S3). The genomic capacity for N_2_ fixation is nearly ubiquitous, as nitrogenase genes (*nif*DHK*)* are present in all binned genomes except *Rhizobium* MD101, improving growth feasibility under low dissolved nutrient conditions. In Well 10, where nitrate and ammonia are ∼10X more concentrated than Well 3, predicted nitrite/ammonia transporters and full nitrate/nitrite reduction and denitrification pathways in *Rhizobium* MD101 suggest that increased dissolved nitrogen bioavailability reduces the necessity for N_2_ fixation and promotes the ability to use nitrate as a terminal electron acceptor. In Well 3, where nitrate is below detection, the genes for dissimilatory nitrate reduction are found only in *Sulfurospirillum* MD31 and MD32. However, the detection of nitrite transporters and incomplete denitrification pathways (Table 3) suggest the potential for intermediate nitrogenous redox species to accumulate *in situ* as syntrophic electron acceptors (Carlson and Ingraham, 1983) similar to activities observed in geothermal springs (Dodsworth *et al*., 2011) and coastal sediments (Fernandes *et al*., 2010). Metagenomes from CO_2_‐rich Crystal Geyser fluids also revealed genes for anaerobic respiration by nitrate reduction and nitrogen fixation (Emerson *et al*., 2015; Probst *et al*., 2016), which together with our results suggest that these metabolic strategies support biosphere survival under high pCO_2_ in different environments.

Many energy‐generating processes may be coupled to redox reactions involving iron Fe(II/III) or arsenic As(III/V). Thermodynamic models of saline aquifers suggest that scCO_2_‐induced acidity increases available energy for Fe(III)‐reduction (Kirk, 2011). Fe(III) reduction is potentially exploited in Well 10 (0.41 mg/l) by *Rhizobium* MD101, which contains ferric‐chelate reductase and Fe(III) transporters. Arsenic present in Well 10 (2.12 mg/l) may enable dissimilatory arsenate reduction by *Sulfurospirillum* MD102, a trait commonly observed in *Sulfurospirillum* (Stolz *et al*., 1999). Though the lower concentration of arsenic in Well 3 (0.01 mg/l) may limit its involvement in redox coupling, genes for dissimilatory arsenate reductase (*arr*AB) detected in *Oscillibacter* MD35 and *Sulfurospirillum* MD31 raise the possibility that redox reactions facilitating arsenic precipitation may limit its detection by ICP. Arsenate reductase detoxification genes (*ars*C) and efflux pumps (*asr*AB, ACR3) detected in all binned genomes suggests the presence of dissolved arsenic prompts a capacity for redox processing and export.

In anaerobic aqueous systems absent terminal electron acceptors or redox couples, fermentation end products (e.g. butanol, succinate, lactate, etc.) supporting the growth of secondary fermenters and acetogens (Table 3) may enable generation of electron carriers acetate and formate (Hattori *et al*., 2001; Kaden *et al*., 2002; De Bok *et al*., 2004). These simple carbon compounds may in turn be metabolized to produce the broadly useful reductant H_2_ (Morris *et al*., 2013). Annotation of acetate kinase (catalyzing acetate formation from acetyl‐CoA) in all genomes and pyruvate‐formate lyase (catalyzing formate production from pyruvate) in all Well 3 genomes suggest that both acetate and formate may be generated by central carbon metabolism. The presence of the TCA cycle in several taxa (Table 3) indicates the capacity to consume acetate (as acetyl‐CoA), while the presence of formate‐hydrogen lyase genes in *Rhizobium* MD10 and all *Sulfurospirillum* genomes suggests that formate may be oxidized to H_2_ and CO_2_. FeFe hydrogenase in *Desulfovibrio* MD33 may also aid in H_2_ production. Since bioavailable H_2_ can serve as an electron donor for numerous respiratory and CO_2_ fixation pathways including denitrification, sulfate reduction, and the Wood‐Ljungdahl Pathway, its rapid utilization may explain the very low *in situ* H_2_ partial pressures observed at McElmo Dome (Nedwell and Banat, 1981; Morris *et al*., 2013). Low levels of accumulated hydrocarbons (Rabinowitz and Janowiak, 2005) may also serve as reduced carbon substrates, as suggested by the predicted capacity of *Desulfosporosinus* to anaerobically activate monoaromatic hydrocarbons (benzylsuccinate synthase; Supporting Information Fig. S8) to make them available as electron donors. Transporters (Supporting Information Table S6C) annotated across all binned genomes indicate the capacity for diverse organic carbon (sugars, acids, alcohols) substrate uptake that may enable heterotrophic growth.

Despite the lethal effects of scCO_2_ exposure and temperatures >65°C, exploitable niches for microbial growth may have emerged at McElmo Dome, although rates of cell division and nutrient cycling are expected to be diminished relative to surface environments (Lovley and Chapelle, 1995). The capacity for persistence and growth may partially be due to the pH buffering capacity of dissolved carbonate in the dolomitic Leadville Formation. In fact, the two wells (3 and 10) that yielded amplifiable DNA have among the highest reported CO_2_ to water ratios at McElmo Dome (Table 1), suggesting that high CO_2_ content does not preclude recovery of microbial biomass. Previous studies (Oppermann *et al*., 2010; Morozova *et al*., 2011) reveal the resilience of sulfate‐reducing bacteria to high pCO_2_ exposure. Reports of Proteobacteria and Firmicutes as dominant phyla following GCS and at the CO_2_‐venting Crystal Geyser formation, respectively, are also consistent with observed taxa at McElmo Dome (Mu *et al*., 2014; Emerson *et al*., 2015).

As McElmo Dome is considered a natural analog for long‐term GCS systems, the diversity of microbial metabolisms observed at the site sheds light on potential growth strategies following scCO_2_ injection. High incidence of genes for CO_2_ utilization among binned genomes indicates that CO_2_ may be used as a substrate for autotrophic growth and anaplerotic reactions. Because CO_2_ captured from coal combustion is often contaminated with SO_2,_ other sulfur species, NOx, arsenic and hydrocarbons, genome pathway annotations suggest these compounds may also serve as substrates and energy sources for communities that assemble following geogenic or anthropogenic emplacement. Widespread capacity for nitrogen fixation may represent a survival mechanism in response nutrient limitation, highlighting the potential for emplaced microbial communities to be stimulated by nitrogenous compound addition. For example, nutrient solutions bearing urea (CH_4_N_2_O) and calcium have been co‐injected with microbial suspensions to successfully induce carbonate biomineralization of injected CO_2_ as a plume containment strategy (Phillips *et al*., 2016). In the context of GCS management considerations, the taxa and associated genomic content detected within the McElmo Dome system may thus inform the types of *in situ* or introduced microbial diversity and nutrient profiles required to exploit metabolic and geochemical potential for safe, long‐term CO_2_ sequestration.

Genome content in McElmo Dome formation fluids suggests that a microbial ecosystem fueled by inorganic electron donors and nutrients may catalyze carbon cycling through CO_2_ fixation and remineralization of organic matter. Therefore, future studies attempting to model the behavior of scCO_2_ injected for GCS should take into account the potential geochemical and physical effects of the deep biosphere. Though the scope of this study was initially limited to a dolomitic carbonate formation, our results serve as a reference for future studies to compare microbial biosphere content in alternative geologic contexts, including scCO_2_ and near‐critical CO_2_ reservoirs in unbuffered sandstone formations (e.g. Bravo Dome, CO) and from surface venting of near critical CO_2_ reservoirs (e.g. Crystal Geyser). These comparisons will help clarify the extent to which geochemical context is a selective driver for diversity and whether certain taxa are specifically adapted to survive high pCO_2_ conditions associated with scCO_2_ emplacement.

## Experimental procedures

### Collection of formation water and characterization of biomass and geochemistry

Fluid‐gas separators at ten CO_2_ production wells from two areas of the McElmo Dome system (Yellow Jacket and Hovenweep fields) were decanted and filled 15 h prior to sample collection. Approximately 40 l of fluid was collected in acid‐washed carboys from the separators and from a surface pond used as a source of well drilling fluids (‘pond’). Using a peristaltic pump on site, samples were pre‐filtered by a Nucleopore 10 μm filter, concentrated onto Sterivex 0.22 μm filter units and filled with buffer (40 mM EDTA, 750 mM sucrose, 50 mM Tris‐HCl). Filters were shipped on dry ice and stored at −80°C. 100 ml of fluids were also fixed on site in 3.7% formaldehyde for microscopy and 1 liter samples were collected in acid‐washed containers for geochemical analyses. Samples were shipped on ice and stored at 4°C. Fluid pH and total salinity were measured on site using pH strips and a handheld refractometer respectively.

For cell enumeration, formaldehyde‐preserved samples were treated with Syto 9 stain (Life Technologies), collected on 0.22 μm polycarbonate filters (Nucleopore), washed twice with PBS buffer, and mounted on glass slides for visualization by epifluorescent microscopy (Zeiss Axioscope). Cell counts were extrapolated based on sample volume to calculate microbial densities.

Samples were prepared for elemental, sulfur, and chloride analyses by acidification with concentrated nitric acid while raw fluids were submitted for nitrate and ammonium analyses. Soluble element profiles were generated using inductively coupled plasma optical emission spectrometry (ICP‐OES), while chloride, nitrate and ammonium were measured using distinct ICP methods (supplementary methods) on an iCAP 6300 (Thermo Fischer) at the Utah State University Analytical Laboratories (Hill Logan, UT).

### DNA extraction

Nucleic acids were extracted from Sterivex filters using two methods: the MoBio UltraClean Soil DNA Isolation Kit and a hot phenol chloroform method (Crump *et al*., 2003; supplementary methods). When using the MoBio Kit, half filters and 500 μl of removed DNA‐protective buffer were added to bead‐beating tubes prior to initial vortexing, but otherwise followed manufacturer's instructions. DNA concentrations measured by NanoDrop 2000c (Waltham, MA) were near detection limits (<20 ng/μl) or unable to be accurately measured due to sample discoloration.

### Sequencing and analysis of 16S rRNA gene amplicons

Microbial diversity in well fluids and the drilling fluids pond was characterized by 16S rRNA gene amplicon clone libraries and next‐generation Illumina sequencing (detailed protocols in supplementary methods). In brief, clone libraries were constructed by ligating amplicons generated using universal small subunit 16S rRNA gene primers SSU_357_F and SSU_1100_R (Supporting Information Table S1) into TOPO TA pCR4 cloning vectors (Invitrogen). Cloned DNA was amplified, purified and submitted for Sanger sequencing at Genewiz (Cambridge, MA). Templates for Illumina sequencing consisted of (1) community genomic DNA from wells and the pond, (2) vector pCR4 containing a library of SSU_357_F/SSU_1100_R 16S rRNA gene amplicons, (3) a no DNA template control, and (4) an archived false positive (AFP) from a discarded PCR run (i.e. a contaminated PCR negative control) to identify potential background laboratory contamination (Salter *et al*., 2014). After amplifying the V3‐V4 hypervariable regions of the 16S rRNA gene using the universal primer set PE_357_F/PE_806_R (Turner *et al*., 1999; Preheim *et al*., 2013) (Supporting Information Table S1), a modified version of the Preheim *et al*. (2013) protocol was used to add barcodes and Illumina sequencing adaptors. Multiplexed samples were sequenced as paired end reads (300 bp + 300 bp) at the MIT BioMicro Center via Illumina MiSeq (v.3 chemistry).

Demultiplexed MiSeq FASTQ files were processed using the UPARSE pipeline (Edgar, 2013) for quality filtering and trimming (385 bp), merging of paired reads, chimera removal, and operational taxonomic unit (OTU) clustering (>97% sequence identity). Phylogenetic annotation utilized the SILVA aligner (Quast *et al*., 2013) and RDP 16S rRNA gene database sequences (Wang *et al*., 2007). Sample OTUs were screened against AFP OTUs to identify and remove sequences likely to be laboratory contaminants. After QC in CLC Genomics Workbench 7, clone library 16S rRNA gene sequences were chimera‐checked and annotated by the same pipeline as Illumina OTUs. Clone library and Illumina OTU sequences are available in the NCBI Genbank database under accession numbers KY457354‐KY457407 and KY466177‐KY466596 respectively.

Chao1 statistics were calculated using Qiime script *alpha_diversity.py*. Abundance‐weighted, normalized UniFrac (Hamady *et al*., 2009) distances were subjected to principle coordinate analysis (PCoA) to compare OTU distributions between samples. SINA‐aligned OTUs present at ≥1% abundance in Well 3 or 10 were used to construct a bootstrapped (100X), neighbor‐joined phylogenetic tree in CLC Genomics Workbench 7 (visualized in FigTree v1.4.2).

### Metagenome sequencing and analysis

Community genomic DNA from Wells 3 and 10 purified using the MoBio Kit and Phenol Chloroform protocols (Supporting Information Table S2) was prepared by the MIT BioMicro Center for metagenome sequencing using the Nextera XT DNA Library Preparation Kit (Illumina), followed by sequencing as paired end reads (300 bp + 300 bp) via Illumina MiSeq (v.3 chemistry). MiSeq FASTQ files were demultiplexed using custom scripts, then trimmed, quality filtered (Quality limit = 0.05; length >50 bp) and *de novo* co‐assembled using default kmer size (CLC Genomics Workbench 7). Assembled contigs (97% similarity over 80% read length) were subjected to tetranucleotide frequency calculations using a Perl script (Albertsen *et al*., 2013) prior to principal component analyses (PCA) and plotting in R. After open reading frame (ORF) prediction by Metaprodigal, 108 single copy genes were identified using a Hidden Markov Model (Albertsen *et al*., 2013) modified to analyse individual genomes rather than time series data. Percent of detected single copy genes was used to predict genome completeness, consistent with standard methods (e.g. CheckM: http://ecogenomics.github.io/CheckM/). Contigs bearing 16S rRNA or single copy genes were subjected to Blastx (NCBI NR database) and classification using MEGAN 5.0. Taxon affiliations were overlaid on a PCA‐tetranucleotide plot to guide extraction of scaffolds represented by individual microbial genomes (‘crude bin’). To ensure that no scaffolds were misplaced into unrelated taxonomic bins, scaffolds within each extracted ‘crude bin’ were fragmented *in silico* to 1000 bp, subjected to six‐frame sequence translations using Diamond (Buchfink *et al*., 2015) and taxonomically annotated using Blastx (NCBI NR database) followed by assignment in MEGAN 5.0 (Supporting Information Fig. S5). Scaffolds comprised of fragments affiliated with unexpected taxa were then placed in bins based on cluster analyses of tetranucleotide frequency homology. Binning several strains of the same species is discussed in supplementary methods (Supporting Information Fig. S6). The presence of potential sequence contaminants was further reviewed by verifying the taxonomic affiliations of single copy genes, 16S and 23S rRNA genes, and ORFs based on best Blastp hits. Tetranucleotide frequencies for contigs (*in silico* fragmented to 2000 bp) in each bin were subjected to Emergent Self Organizing Map analyses (default settings except K‐Batch training algorithm in 80x120) to visually ascertain contig separation and remove any improperly binned sequences. The number of metagenomic raw reads assigned to each genomic bin was used to calculate relative taxonomic abundances. Each genomic bin was submitted to RAST and IMG for automated functional annotation. Community metagenome sequences for Wells 3 and 10 are deposited the IMG database (https://img.jgi.doe.gov) under Genome IDs 3300006428 and 3300006427, respectively, and individual extracted genomes under IDs 2639762641‐2639762647.

## Disclaimer

This publication was prepared as an account of work sponsored by an agency of the United States Government. Neither the United States Government nor any agency thereof, nor any of their employees, makes any warranty, express or implied, or assumes any legal liability or responsibility for the accuracy, completeness, or usefulness of any information, apparatus, product, or process disclosed, or represents that its use would not infringe privately owned rights. Reference herein to any specific commercial product, process, or service by trade name, trademark, manufacturer, or otherwise does not necessarily constitute or imply its endorsement, recommendation, or favoring by the United States Government or any agency thereof. The views and opinions of authors expressed herein do not necessarily state or reflect those of the United States Government or any agency thereof.

## Conflict of Interest Statement

We the authors confirm that this manuscript has not been published elsewhere and is not under consideration by another journal.

## Supporting information

Additional Supporting Information may be found in the online version of this article at the publisher's website:

**Fig. S1.** At right, location of McElmo Dome system within the Colorado Plateau in SW Colorado. Inset, approximate well cluster fluid sampling locations in the Hovenweep (green) and Yellow Jacket (blue) fields and drilling fluids pond (red). Adapted from Gilfillan et al. (2008).
**Fig. S2. (A)** Hierarchical clustering by Spearman rank correlation of sample ICP‐OES profiles. Heat map displays log transformed (X+1) mg/l concentrations. Clustering reveals three geochemical signature groups. **(B)** Base 10 normalized CO2 and H2O well test values.
**Fig. S3.** Rarefaction curve generated for initial OTU table based on raw reads demonstrates a sampling of system diversity that nears completion for most wells, the drilling fluids pond and AFP control.
**Fig. S4.** Phylogenetic tree of Illumina‐sequenced 16S rRNA gene OTUs from McElmo Dome at great than 1% abundance in Wells 3 and/or Well 10 displaying the phylum and genus level RDP/Silva annotations. SINA‐aligned sequences were constructed into a neighbor‐joined, bootstrapped (100) tree in CLC Genomics Workbench 7, and visualized FigTree. Tree rooted on outgroup Archaeal species *Nitrosopumilus maritmus*.
**Fig. S5.** Sequence coverage and GC content of contigs in Well 3 that could not be separated based on tetranucleotide frequencies and sequence homology. Each dot represents a single scaffold/contig with minimum contig length of 1000 bp. All contigs with sequence coverage lower than 40 were fragmented to 500 bp in silico followed by Blastx searches against the NCBI NR‐database and subsequently assigned a taxon using MEGAN with bitscore of 100. Contigs containing fragments with hits to a single taxon were classified to the same taxonomic group unambiguously (i.e. *Acetobacterium*, *Desulfosporosinus*, *Peptococcaceae* and *Bacteroides*) and plotted to guide extraction of genomic bin.
**Fig. S6.** Psi‐Blast comparison of the ORFs between binned *Sulfurospirillum* genomes and reference genome *S. deleyanium* DSM 6946 using RAST default settings.
**Fig. S7.** Maximum likelihood tree of reference dsrAB amino acid sequences together with full‐length dsrAB recovered from Metagenome 3. A HMM model for dsrAB was used in screening individual genomic bins and complete Metagenomes 3 and 10. No dsrAB was detected in Metagenome 10. The phylogenetic tree was constructed with 1000X bootstrapping and rooted using pyruvate formate lyase gene in *Clostridium noyvi* (WP_039252367). Bootstrap support values >60 are shown on each branch.
**Fig. S8.** Maximum likelihood tree of reference AssA/BssA amino acid sequences together with full‐length bssA (bold) recovered from Metagenome 3. A HMM model for AssA and BssA was used in screening individual genomic bins and complete Metagenomes 3 and 10. No AssA/BssA was detected in Metagenome 10. The phylogenetic tree was constructed with 1000X bootstrapping and rooted using pyruvate formate lyase gene in *Clostridium noyvi* (WP_039252367). Bootstrap support values >60 are shown on each branch.
**Fig. S9.** Maximum likelihood tree of reference Ribulose 1,5‐bisphosphate (RuBP) carboxylase/oxygenase (RuBisCO) amino acid sequences together with RuBisCO gene (Red) recovered from Well 10 binned genome *Rhizobium* MD101. Phylogenetic tree was constructed with 100X bootstrapping. Clustered sequences listed in Table S7.

**Table S1.** Summary of pcr primers used in clone library and Illumina sequencing preparation.
**Table S2.** Sample preparation methods for Illumina sequencing.
**Table S3.** ICP‐OES analyte summary for major elements in McElmo Dome formation.
**Table S4.** Clone library taxonomic annotation and abundance summary.
**Table S5.** Distribution of Archived False Positive (AFP) OTUs discarded as contaminants from samples.
**Table S6.** RAST/IMG‐annotated genes associated with **A**) inorganic metabolic pathways and cycling, **B**) organic metabolic pathways and hydrogenases and **C**) membrane transporters.
**Table S7.** Reference RuBisCO sequences plotted in Figure S9.

Supplementary Methods
