# Supplementary material for: Microbial potential for carbon and nutrient cycling in a geogenic supercritical carbon dioxide reservoir
Source: Environ Microbiol. 2017 May 2;19(6):2228–45. doi: 10.1111/1462-2920.13706 (PMC5518199; doi:10.1111/1462-2920.13706)
Supplement: Supplementary file 1 — Fig. S1. At right, location of McElmo Dome system within the Colorado Plateau in SW Colorado. Inset, approximate well cluster fluid sampling locations in the Hovenweep (green) and Yellow Jacket (blue) fields and drilling fluids pond (red). Adapted from Gilfillan et al. (2008). Fig. S2. (A) Hierarchical clustering by Spearman rank correlation of sample ICP‐OES profiles. Heat map displays log transformed (X+1) mg/l concentrations. Clustering reveals three geochemical signature groups. (B) Base 10 normalized CO2 and H2O well test values. Fig. S3. Rarefaction curve generated for initial OTU table based on raw reads demonstrates a sampling of system diversity that nears completion for most wells, the drilling fluids pond and AFP control. Fig. S4. Phylogenetic tree of Illumina‐sequenced 16S rRNA gene OTUs from McElmo Dome at great than 1% abundance in Wells 3 and/or Well 10 displaying the phylum and genus level RDP/Silva annotations. SINA‐aligned sequences were constructed into a neighbor‐joined, bootstrapped (100) tree in CLC Genomics Workbench 7, and visualized FigTree. Tree rooted on outgroup Archaeal species Nitrosopumilus maritmus. Fig. S5. Sequence coverage and GC content of contigs in Well 3 that could not be separated based on tetranucleotide frequencies and sequence homology. Each dot represents a single scaffold/contig with minimum contig length of 1000 bp. All contigs with sequence coverage lower than 40 were fragmented to 500 bp in silico followed by Blastx searches against the NCBI NR‐database and subsequently assigned a taxon using MEGAN with bitscore of 100. Contigs containing fragments with hits to a single taxon were classified to the same taxonomic group unambiguously (i.e. Acetobacterium, Desulfosporosinus, Peptococcaceae and Bacteroides) and plotted to guide extraction of genomic bin. Fig. S6. Psi‐Blast comparison of the ORFs between binned Sulfurospirillum genomes and reference genome S. deleyanium DSM 6946 using RAST default settings. Fig. S7. Maximu [file EMI-19-2228-s001.pdf]

Figure S1

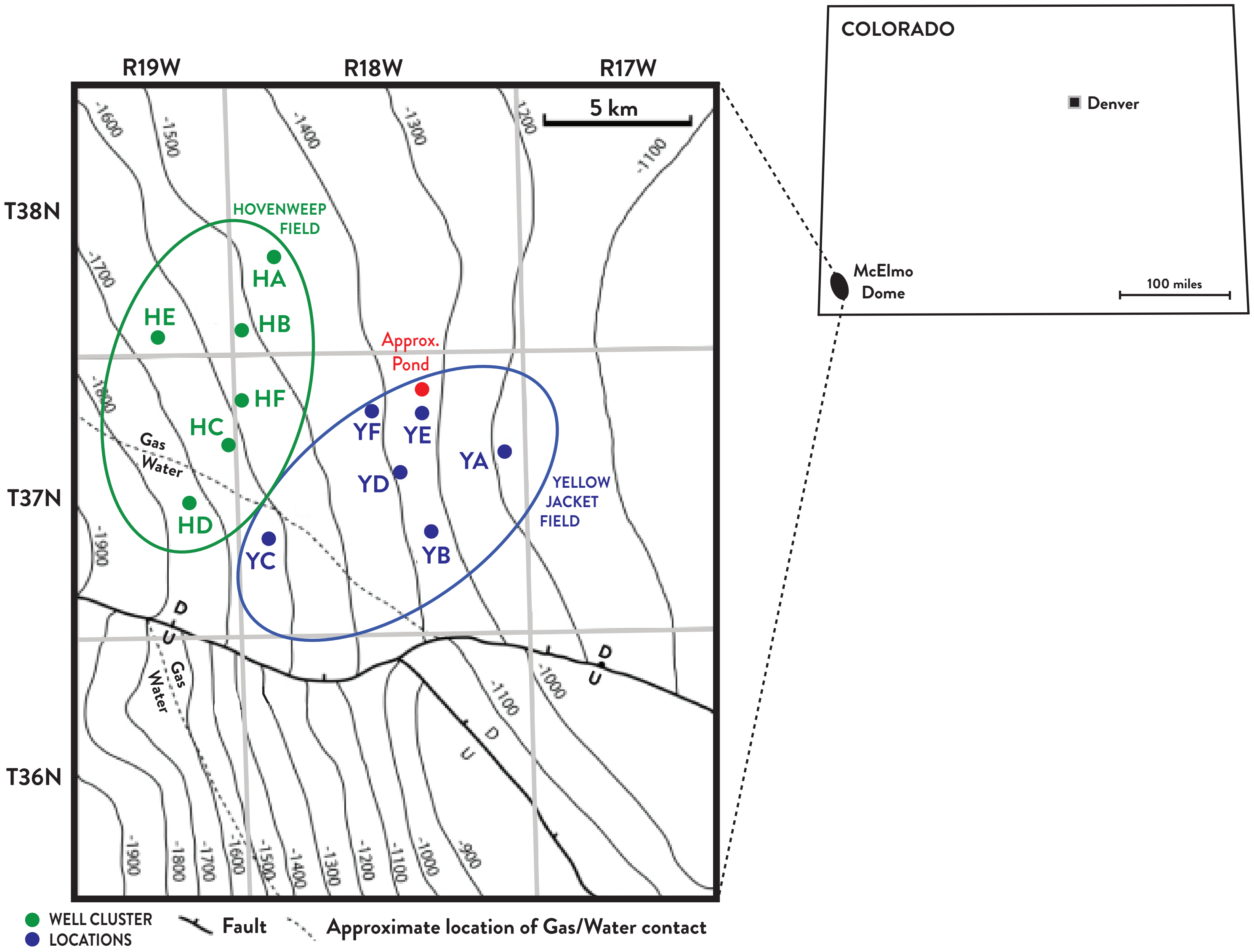

Figure S1. At right, location of McElmo Dome system within the Colorado Plateau in SW Colorado. Inset, approximate well cluster fluid sampling locations in the Hovenweep (green) and Yellow Jacket (blue) fields and drilling fluids pond (red). Adapted from Gilfillan et al., 2008.

Figure S2

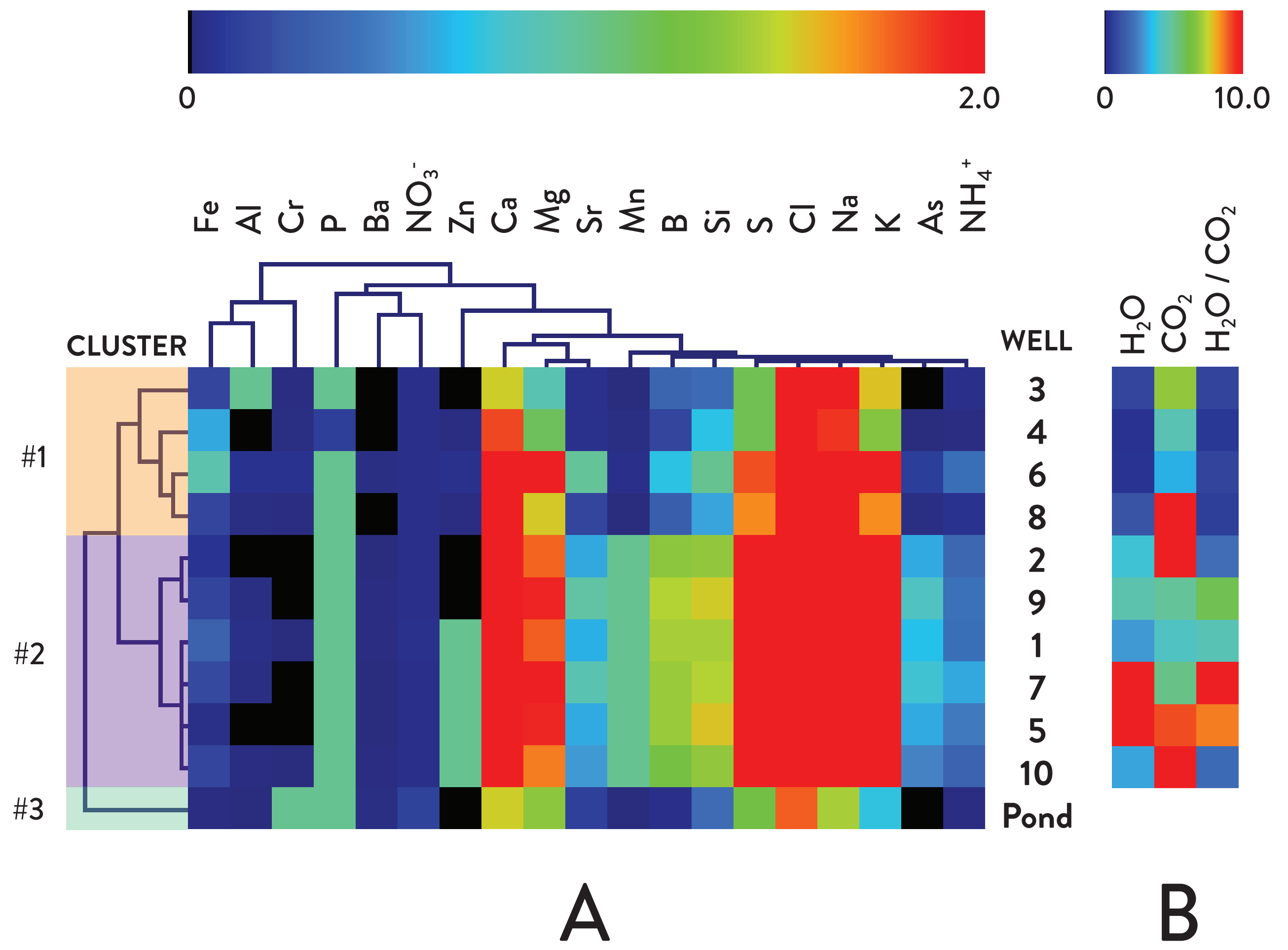

Figure S2. A) Hierarchical clustering by Spearman rank correlation of sample ICP-OES profiles. Heat map displays log transformed (X+1) mg/l concentrations. Clustering reveals three geochemical signature groups. **B)** Base 10 normalized CO_2 and H_2O well test values.

Figure S3

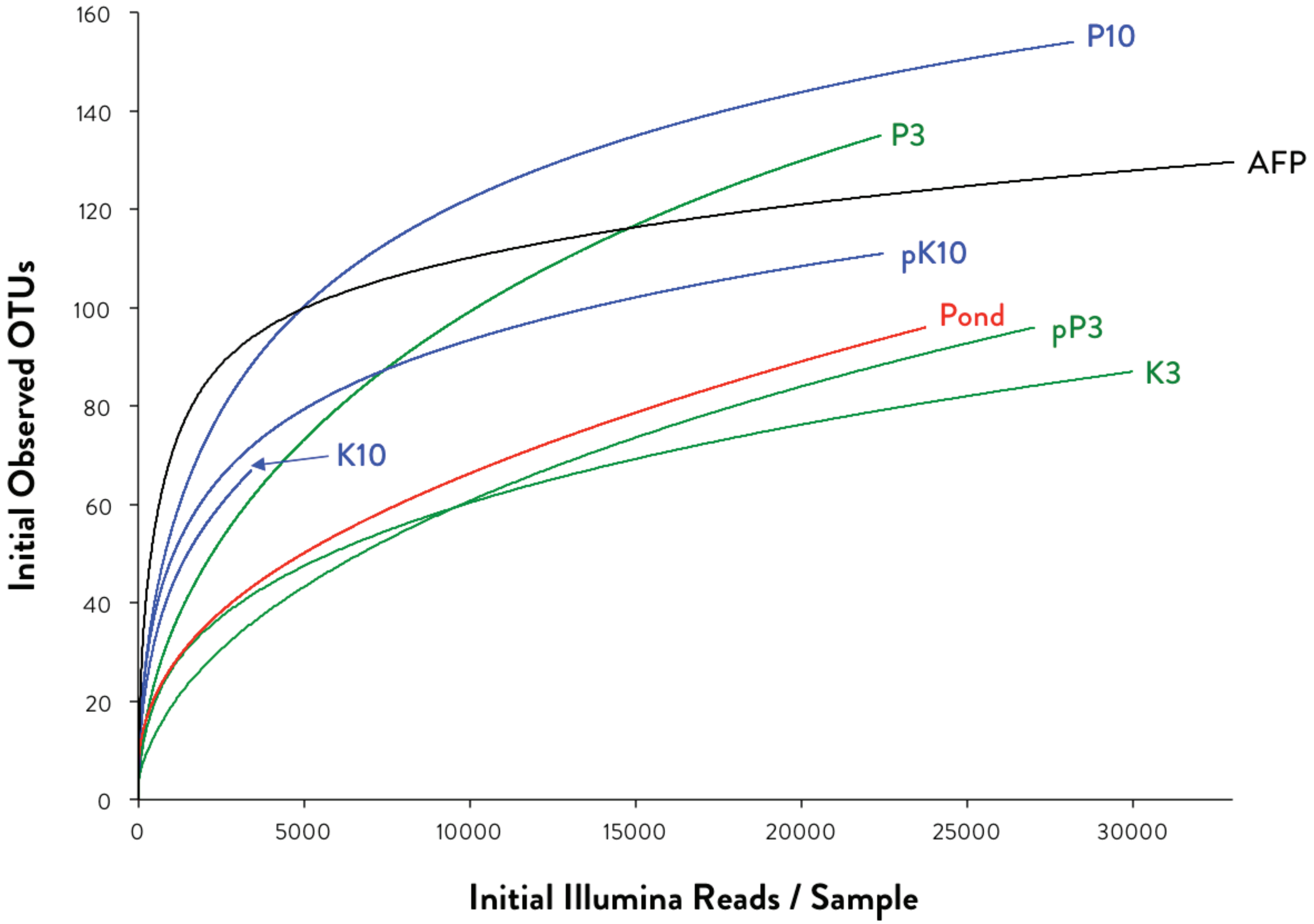

Figure S3. Rarefaction curve generated for initial OTU table based on raw reads demonstrates a sampling of system diversity that nears completion for most wells, the drilling fluids pond and AFP control.

Figure S4

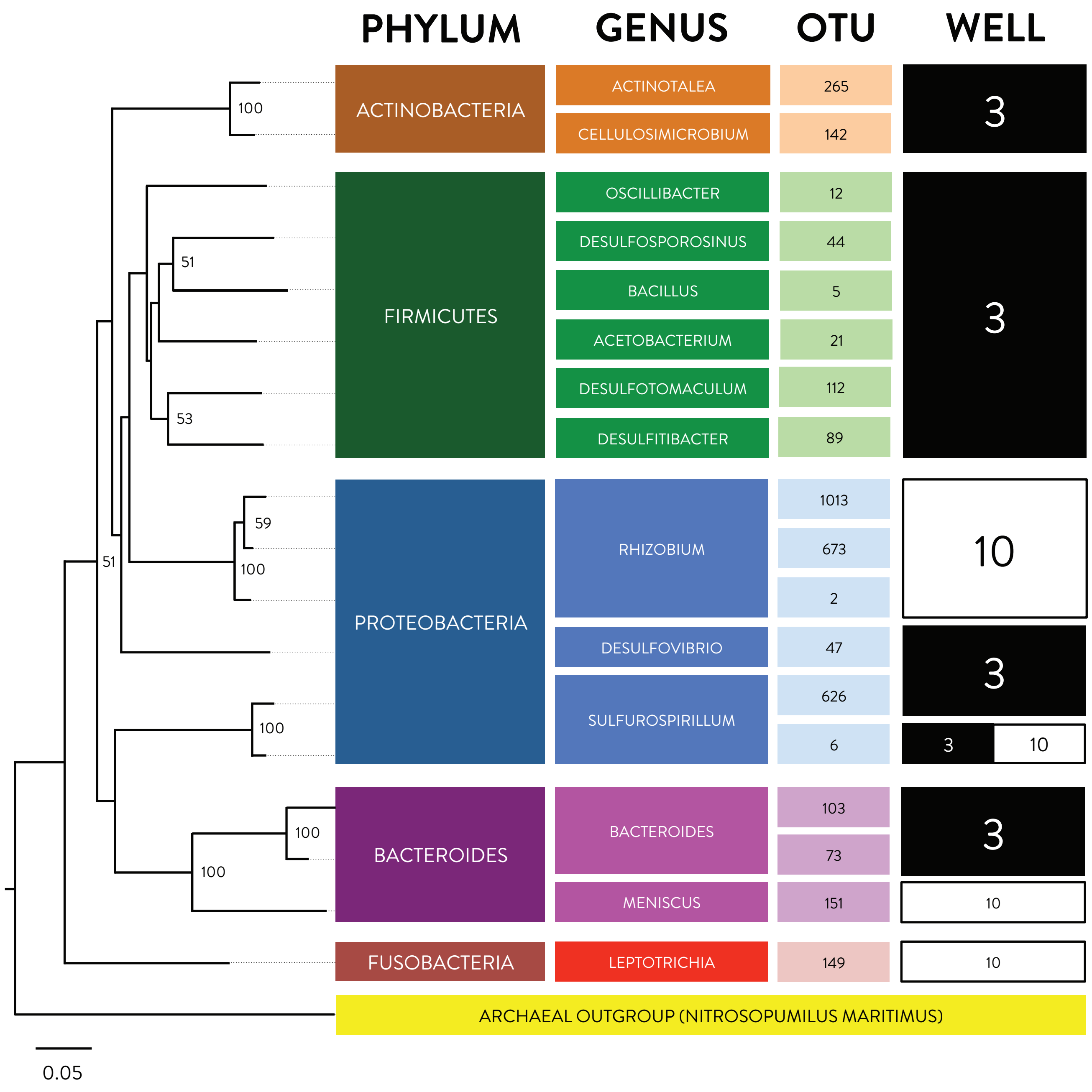

Figure S4. Phylogenetic tree of Illumina-sequenced 16S rRNA gene OTUs from McElmo Dome at great than 1% abundance in Wells 3 and/or Well 10 displaying the phylum and genus level RDP/Silva annotations. SINA-aligned sequences were constructed into a neighbor-joined, bootstrapped (100) tree in CLC Genomics Workbench 7, and visualized FigTree. Tree rooted on outgroup Archaeal species *Nitrosopumilus maritimus*.

Figure S5

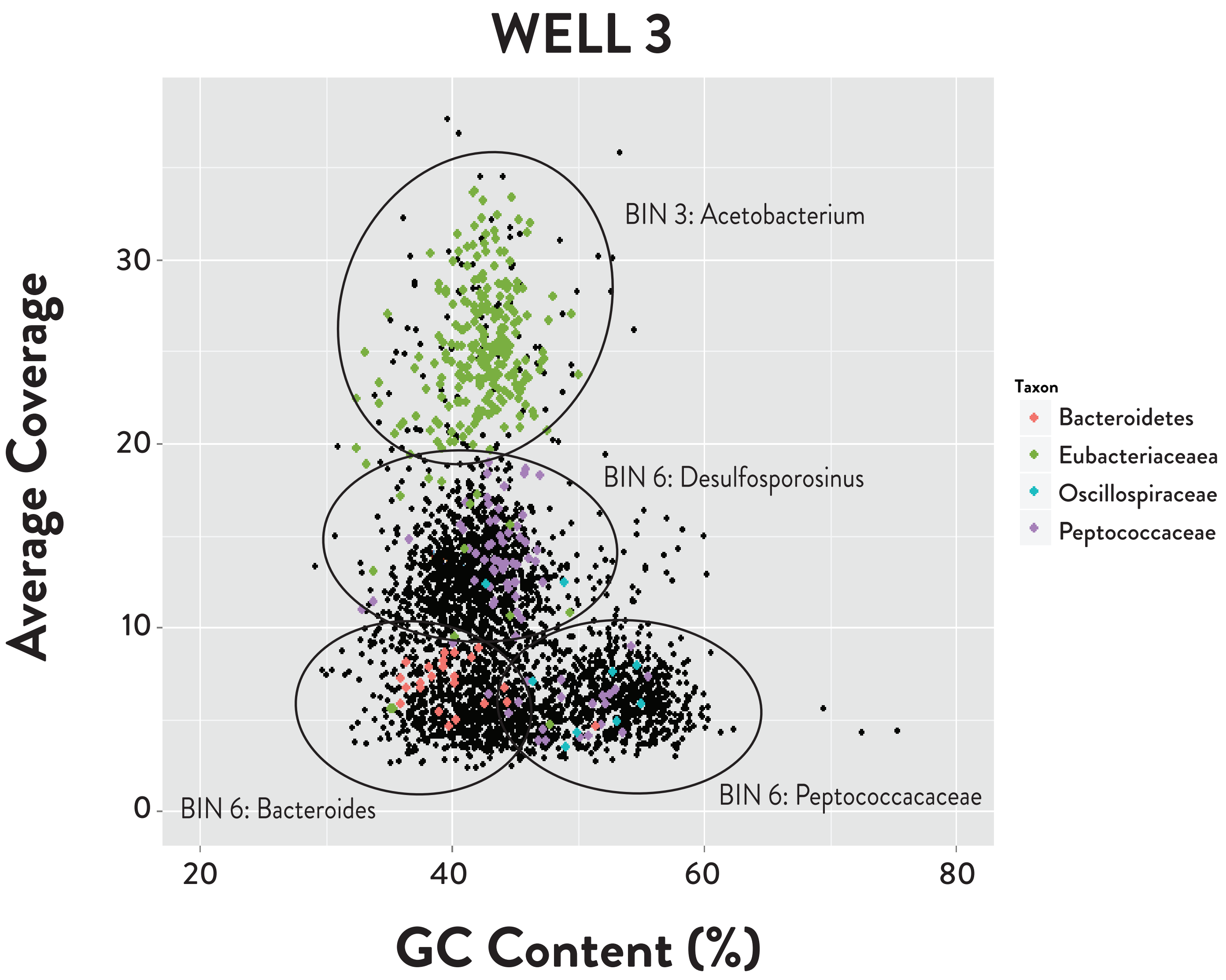

Figure S5. Sequence coverage and GC content of contigs in Well 3 that could not be separated based on tetranucleotide frequencies and sequence homology. Each dot represents a single scaffold/contig with minimum contig length of 1000 bp. All contigs with sequence coverage lower than 40 were fragmented to 500 bp in silico followed by Blastx searches against the NCBI NR-database and subsequently assigned a taxon using MEGAN with bitscore of 100. Contigs containing fragments with hits to a single taxon were classified to the same taxonomic group unambiguously (i.e. *Acetobacterium*, *Desulfosporosinus*, *Peptococcaceae* and *Bacteroides*) and plotted to guide extraction of genomic bin.

Figure S6

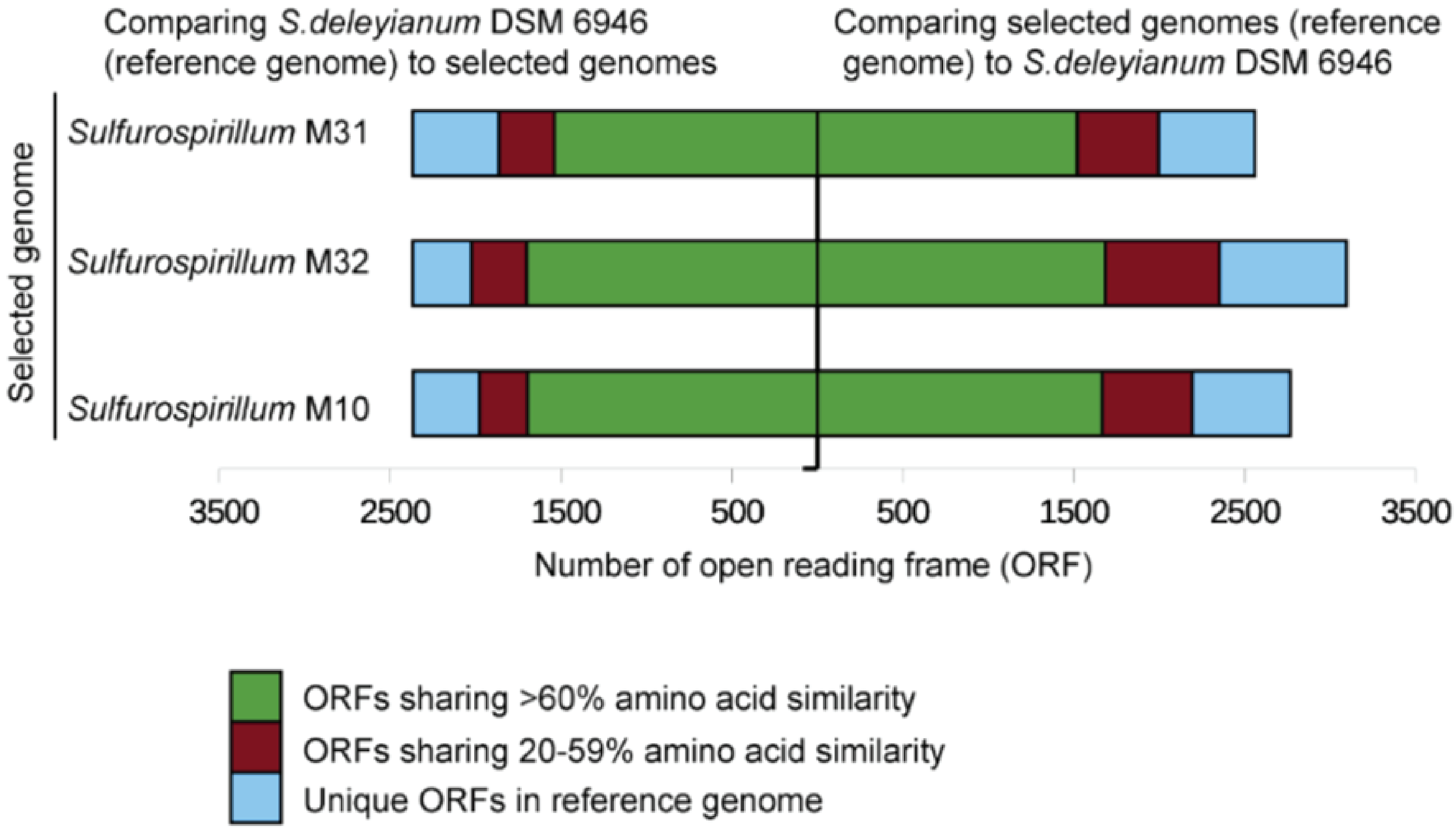

Figure S6. Psi-Blast comparison of the ORFs between binned *Sulfurospirillum* genomes and reference genome *S. deleyanium* DSM 6946 using RAST default settings.

Figure S7

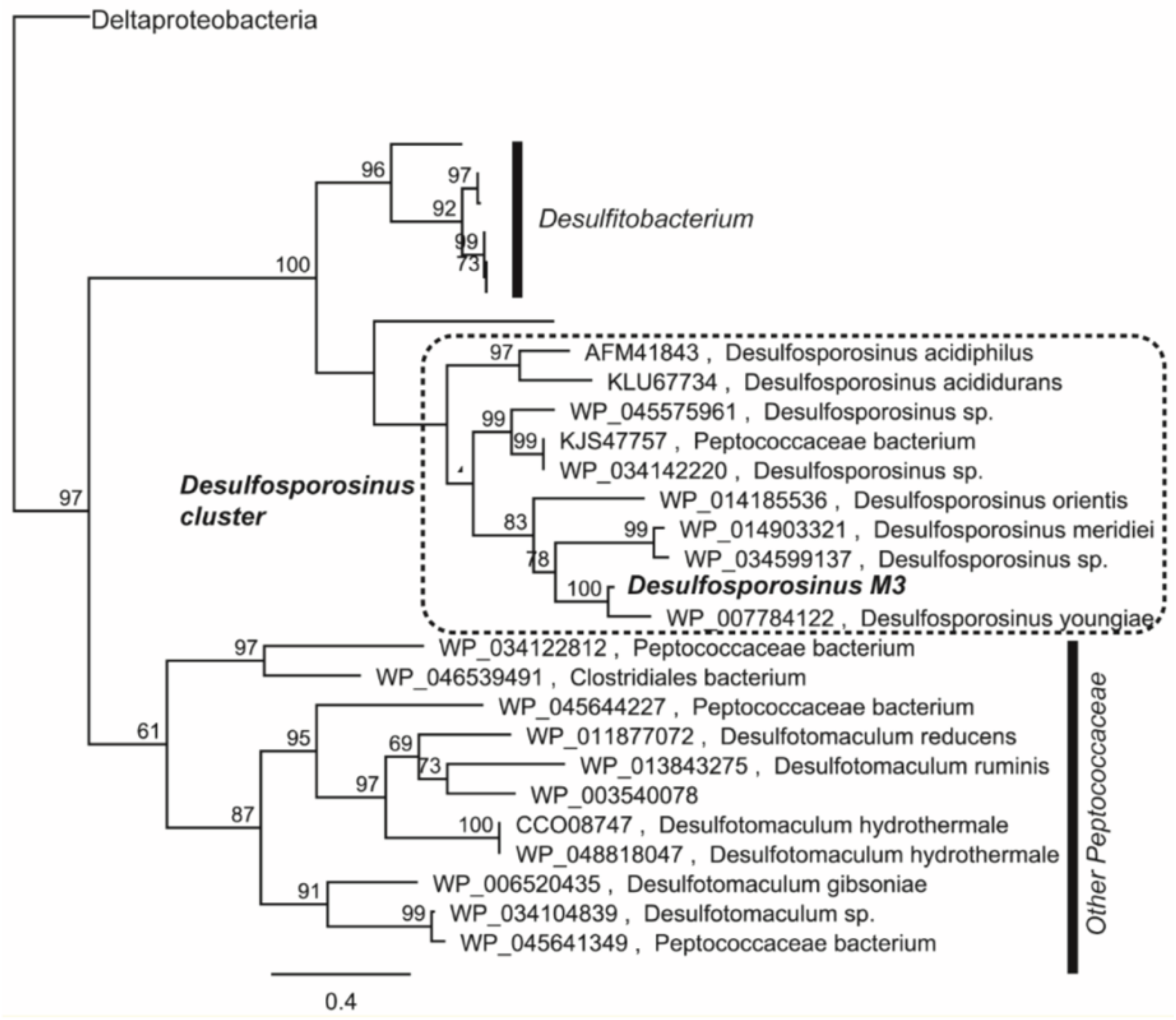

Figure S7. Maximum likelihood tree of reference dsrAB amino acid sequences together with full-length dsrAB recovered from Metagenome 3. A HMM model for dsrAB was used in screening individual genomic bins and complete Metagenomes 3 and 10. No dsrAB was detected in Metagenome 10. The phylogenetic tree was constructed with 1000X bootstrapping and rooted using pyruvate formate lyase gene in *Clostridium noyvi* (WP_039252367). Bootstrap support values >60 are shown on each branch.

Figure S8

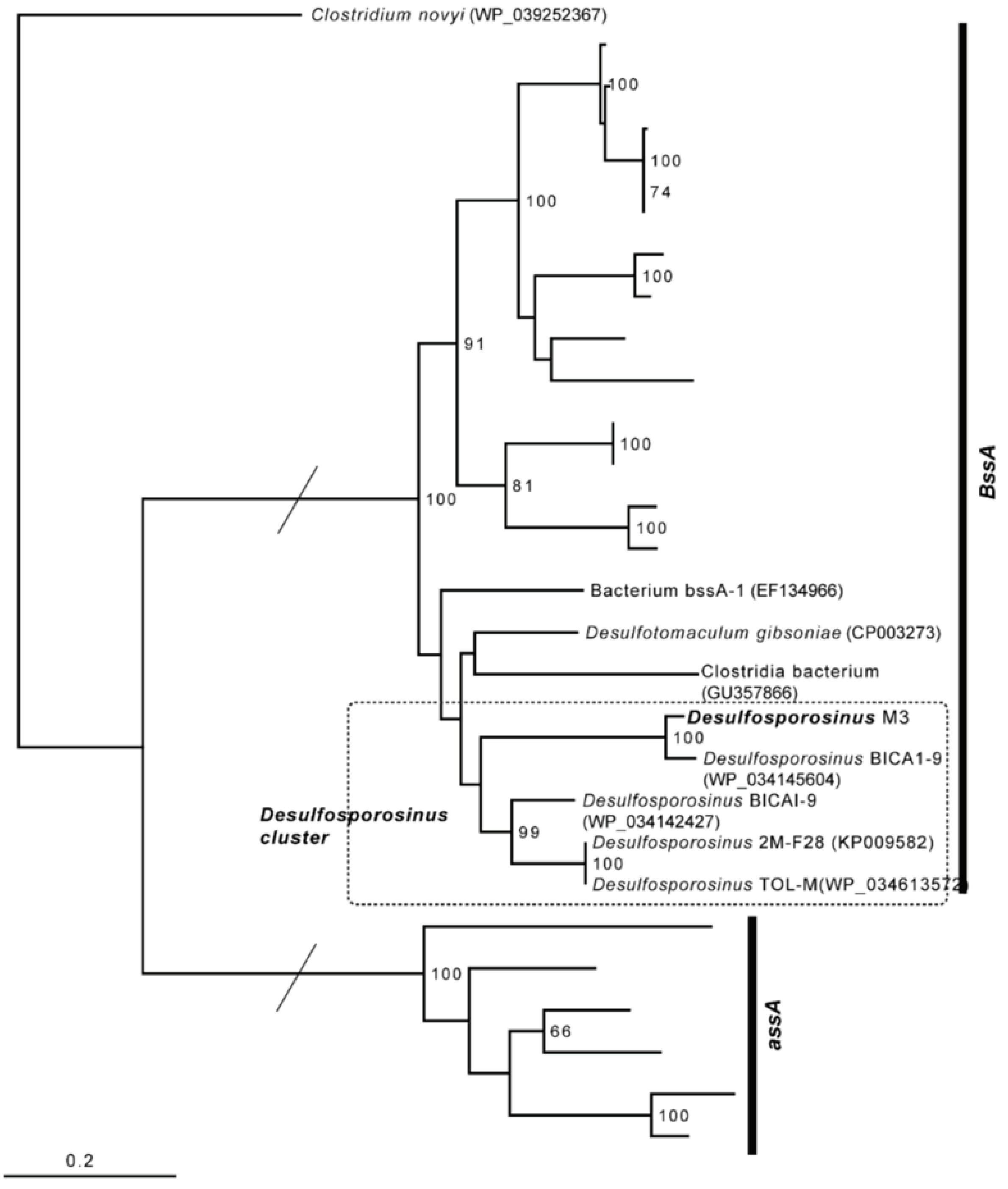

Figure S8. Maximum likelihood tree of reference AssA/BssA amino acid sequences together with full-length bssA (bold) recovered from Metagenome 3. A HMM model for AssA and BssA was used in screening individual genomic bins and complete Metagenomes 3 and 10. No AssA/BssA was detected in Metagenome 10. The phylogenetic tree was constructed with 1000X bootstrapping and rooted using pyruvate formate lyase gene in *Clostridium noyvi* (WP_039252367). Bootstrap support values >60 are shown on each branch.

Figure S9

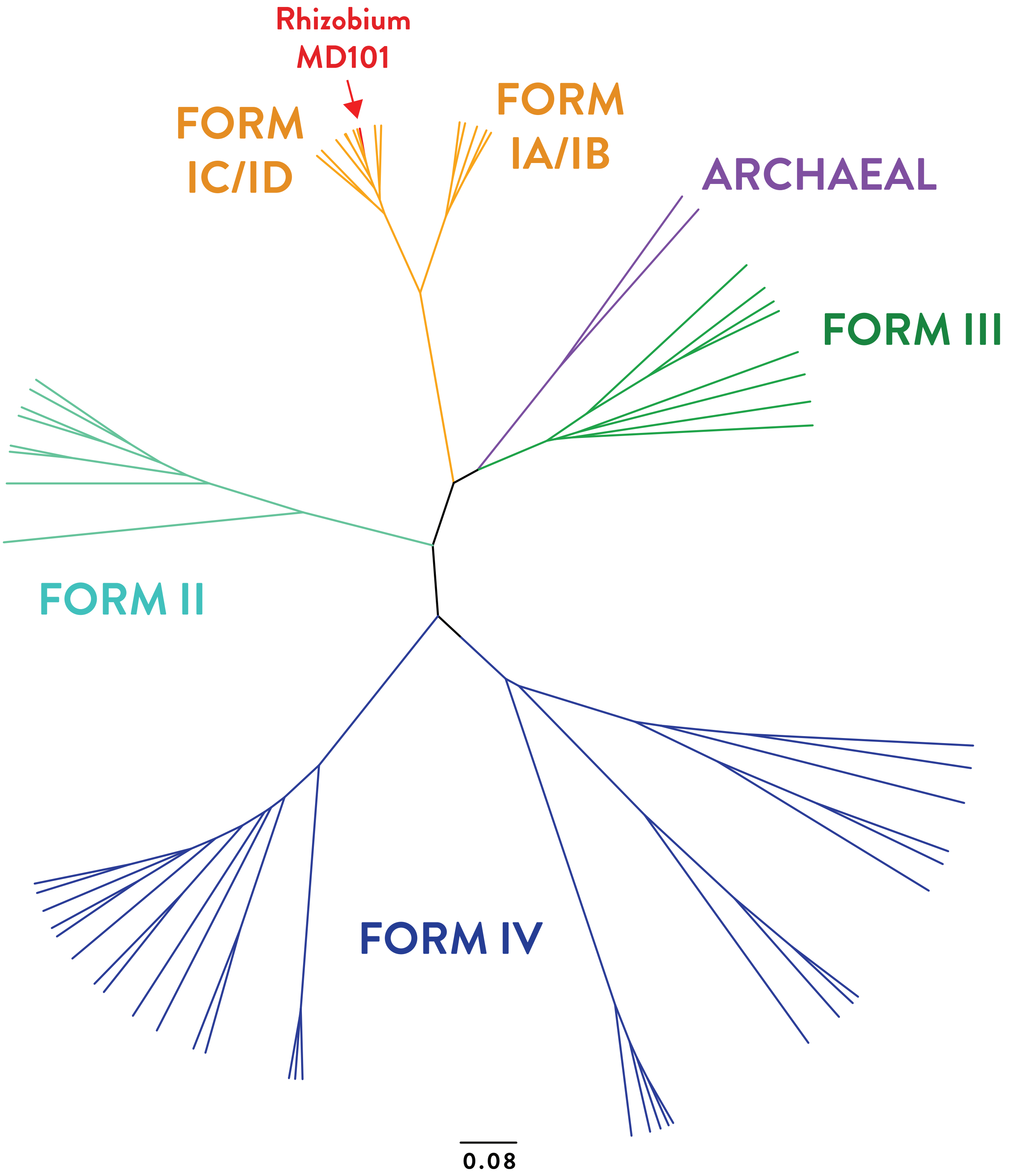

Figure S9. Maximum likelihood tree of reference Ribulose 1,5-bisphosphate (RuBP) carboxylase/oxygenase (RuBisCO) amino acid sequences together with RuBisCO gene (Red) recovered from Well 10 binned genome *Rhizobium* MD101. Phylogenetic tree was constructed with 100X bootstrapping. Clustered sequences listed in Table S7.
