# Supplementary material for: Microbial potential for carbon and nutrient cycling in a geogenic supercritical carbon dioxide reservoir
Source: Environ Microbiol. 2017 May 2;19(6):2228–45. doi: 10.1111/1462-2920.13706 (PMC5518199; doi:10.1111/1462-2920.13706)
Supplement: Supplementary file 3 — Supplementary Methods [file EMI-19-2228-s003.docx]

**Supplementary Methods**

**Site Characterization**

476 billion m^3^ of CO_2_ in the 800 km^2^ McElmo Dome field began to accumulate approximately 40 to 72 million years ago (Cappa and Rice, 1995; Gilfillan *et al*., 2008). Trapped at depths of 1800 to 2600 m within the 100 m thick dolomite-rich Leadville Formation (Allis *et al*., 2001; Gilfillan *et al*., 2009), CO_2_ exists as a supercritical fluid above the CO_2_ critical point (>31°C, 71 atm) at the temperature (~65°C) and pressure (~135 atm) conditions of the formation. KinderMorgan operates 61 wells in the Leadville Formation that produce fluids with a wide range of CO_2_/H_2_O ratios, from pure CO_2_ to nearly degassed samples. On site, an artesian mixture of supercritical CO_2_ and saline formation water is produced and separated, after which the CO_2_ is further dehydrated and compressed for pipeline delivery and commercial use, while the formation water is re-injected into the Leadville Formation (Stevens *et al*., 2001).

**Geochemical Analysis Methods**

Chloride, Nitrate and Ammonium flow-injection analyses were conducted using a Thermo iCAP6300 ICP system with colorimetric determinations. The specific methods include, for chloride: Lachat QuikChem Method 10-117-07-1-C (FIA); for nitrate: Lachat QuickChem Method No. 12-107-04-1-B, using a cadmium-reduction column; and for ammonium: Latchat QuickChem Method No. 12-107-06-1-B.

**DNA Extraction Methods**

DNA-protective buffer was removed from Sterivex filters by a sterile 1 ml syringe. A hammer was then used to gently crack open Sterivex cartridges. Using a sterile razor blade, filter membranes were then removed, cut into halves, and added to screw cap tubes with ~0.25 g of sterile 0.1 mm zirconium beads, 0.25 mg/ml proteinase K, and 1 mg/ml lysozyme. Tubes were bead beat at 3000 rpm for 30 sec. Tubes were incubated at 55°C for 20 min, then 70°C for 5 min to inactivate enzymes. The solution was removed from tube, and added to 1 ml phenol:chloroform:isoamyl alcohol (25:25:1), inverted to mix, and incubated at 55°C for 5 minutes. The mixture was then centrifuged at 13,000 rpm for 10 min to separate phases, at which point the aqueous phase was transferred to a fresh tube. The mixture-incubation-phase separation steps were then repeated, and the aqueous phase decanted. The same steps were then repeated, but using 500 μl of pure chloroform. DNA was then precipitated with 10% 3M sodium acetate and 0.6 volumes of isopropanol. The mixture was then incubated overnight at -20°C. The next day, the mixture was centrifuged at 13,000 rpm for 10 min, supernatant removed, washed in 1 ml 70% ethanol, and centrifuged again at 13,000 rpm for 10 min. The supernatant was removed, and the pellet was resuspended in 50 μl of TE buffer, pH 8. The mixture was then allowed to sit overnight at 4°C, before being centrifuged down to trap any condensation. The extracted DNA solution was then stored at -20°C until use.

**Preparation and sequencing of 16S rRNA gene clone libraries**

Community genomic DNA extracted from each of the 10 wells, drilling mud source pond, and controls for potential laboratory contamination, consisting of DNA preservation buffer and MilliQ water, were PCR amplified with universal Bacterial small subunit 16S rRNA 357 forward (SSU_357_F) and 1100 reverse (SSU_1100_R) primers using *Taq* DNA Pol (New England Biolabs) to target the V3-V5 hypervariable regions of the 16S rRNA gene. All primer sequences are listed in Table S1. Final primer concentrations were 0.18 μM. Template, MgCl_2_, and bovine serum albumin (BSA) concentrations were optimized to reduce impacts from PCR inhibition. As a result, the final PCR Master Mix included a final concentration of 1.88 μM MgCl_2_ (including buffer MgCl_2_ content) and 1.76 mg/ml BSA (New England Biolabs). 357F/1100R amplification was performed on the Veriti 96 Well Thermal Cycler (Applied Biosystems) using the following cycling conditions: initial denaturation at 95°C for 3 min, thirty cycles of denaturation at 95°C for 30 sec, annealing at 52°C for 30 sec and extension at 72°C for 1 min, before a final extension at 72°C for 7 min. PCR amplicons were ligated and transformed using the TOPO TA pCR4 cloning kit (Invitrogen, Carlsbad, CA) according to the manufacturer’s instructions. Transformed colonies were used as PCR template with M13 primers, generating amplicons that were gel purified (QIAquick Gel Extraction Kit, Qiagen) and submitted for Sanger sequencing at Genewiz (Cambridge, MA).

**Illumina MiSeq sample library preparation**

All templates were amplified with primers PE_357_F and PE_806_R using Phusion High Fidelity Polymerase according to the following cycling conditions: initial denaturation at 96°C for 1:30 min, thirty cycles of denaturation at 96°C for 30 sec, annealing at 52°C for 30 sec, and extension at 72°C for 30 sec, followed by a final extension at 72°C for 5 min. After PCR amplification, amplicons were purified using Exo-SAP IT (Affymetrix) according to manufacturer’s instructions. 1 μl of purified product was used as template in the subsequent Illumina adaptor addition and barcoding PCR cycle, using universal forward primer PE_SEQ_F and six nucleotide barcode-specific reverse primers PE_SEQ_R at final concentrations of 0.5 μM each, according to the following cycling conditions: initial denaturation at 96°C for 1:30 min, fifteen cycles of denaturation at 96°C for 30 sec, annealing at 65°C for 30 sec, and extension at 72°C for 30 sec, followed by a final extension at 72°C for 5 min. Barcodes were designed with three base differences between any two barcodes. The amplicons were loaded on a 2.0% agarose electrophoresis gel and run at 90V for 60 minutes. DNA bands of the appropriate size were excised from the gel using individual UV-treated scalpels and purified using the QIAquick Gel Extraction Kit (Qiagen). Purified barcoded samples were eluted in 30 μl DEPC water. The relative concentration of each barcoded sample was determined by qPCR. Each sample was used as template in triplicate 20 μl reactions using Illumina sequencing primers (0.5 μM final concentration) and 1X fluorescent Sybr. Samples were multiplexed in volumetric proportions based on minimum cycle number in order to enable equal reads per sample, following Preheim et al. (2013). Pooled samples were concentrated using the MinElute Reaction Cleanup Kit (Qiagen), eluted in 11 μl of DEPC water.

**16S rRNA clone library sequence processing**

Clone library Sanger sequences were subjected to quality filtering and trimming using CLC Genomics Workbench 7. Sequences with quality scores >0.05 or fewer than 400 bases were excluded from analysis. Chimeras were removed using the UPARSE command *uchime_ref* with the gold.db ChimeraSlayer database (Broad Microbiome Utilities). Sequences were annotated using the RDP classifier (Wang *et al*., 2007) and Silva 16S database (Quast *et al*., 2013).

**16S rRNA Illumina sequence processing, OTU clustering and annotation**

Paired-end sequences were merged (fastq_mergepairs) and filtered by designated overlap requirements (fastq_truncqual 3; fastq_minovlen 16). Merged pairs were then mapped using CLC Genomic Workbench to the PhiX virus genome (used by Illumina as an internal sequencing control). PhiX-mapped sequences were removed from downstream analysis. Merged pairs were then filtered according to the recommended expected error threshold (fastq_maxee 3.0; Robert Edgar, Personal Communication). Filtered merged pairs were then trimmed to a length of 385 bases (fastq_filter; fastq_trunclen 385). Sequences shorter than 385 bp were discarded. Trimmed sequences were dereplicated (derep_fulllength) and annotated by number of replicate reads per sequence (sortbysize). All singleton sequences were discarded (minsize 2), per recommended settings. Remaining sequences were then clustered to form OTUs (cluster_otus) at a 97% minimum identity threshold. After chimeric OTU were removed (uchime_ref), merged paired-end reads were then mapped to OTUs using the USEARCH algorithm (usearch_global) at a 97% minimum identity threshold. Readmaps for each sample were then converted into OTU tables using the python script *uc2otutab.py*. OTUs were taxonomically annotated by the python script *assign_taxonomy.py*, using the RDP classifier (Wang *et al*., 2007) and Silva 16S database (Quast *et al*., 2013) with a minimum 60% identity RDP confidence threshold. OTUs that displayed less than 60% confidence to any RDP assignment on the phylum level were discarded (n = 6). Remaining OTUs annotated as the PhiX virus (used as MiSeq internal standard) were removed from downstream analysis. All 16S rRNA OTU sequences are deposited in the NCBI Genbank database with accession numbers to be provided.

**Binning of unique *Sulfurospirillum* (MD31, MD32, MD102) genomes**

Binning of contigs in Metagenome 3 yielded a single genomic bin (63 contigs/5.9 Mbp) containing two strains of *Sulfurospirillum* (based on total number and best Blastp hits of single copy genes (214 copies, assuming a single genome contains roughly 107 genes) and presence of two copies of non-overlapping 16S rRNA gene fragments). This finding is consistent with clustering of 16S rRNA amplicons (97%), which yielded two OTUs affiliated with *Sulfurospirillum* in which one OTU is more dominant than the other (~0.1%). Attempts to separate the two strains of *Sulfurospirillum* (63 contigs; 5.9 Mbp) using GC content (32-48%), tetranucleotide frequencies and contig coverage were unsuccessful. These contigs displayed comparable coverage (~150X) likely because high genome similarity made mapping of short reads indiscriminate. Blastn comparison between the 16S rRNA gene of *Sulfurospirillum* sp. MD102 (single copy of 1431 bp) and the two copies detected in the two strains of *Sulfurospirillum* in Metagenome 3 (fragment of 676 bp of 1088 bp contig (*Sulfurospirillum* MD32) and 523 bp of 1605 bp (*Sulfurospirillum* MD31) aligned partially with that in *Sulfurospirillum* MD102 revealed that one partial fragment (contig 442 in MD31) of the two 16S rRNA genes is 100% similar (overlapping region of 523 bp in MD31) with that in *Sulfurospirillum* MD102, while the second copy has 99% similarity with *Sulfurospirillum* MD32. Due to the similarity of 16S rRNA gene and results from clustering of 16S rRNA gene amplicon showing that MD10 and Metagenome 3 share a single dominant OTU affiliated with *Sulfurospirillum*, all 62 contigs from Metagenome 3 *Sulfurospirillum* bins were subjected to Blastn comparison against all 590 contigs in *Sulfurospirillum* MD102. Contigs in *Sulfurospirillum* MD31 having 100% nucleotide sequence similarity with at least 300 bp overlapping region with MD102 were preliminarily grouped in one genomic bin (*Sulfurospirillum* MD31; 34 contigs) with the remaining contigs assigned to *Sulfurospirillum* MD32 (29 contigs). Several instances suggest that this approach has separated the two strains of *Sulfurospirillum* into their proper respective bins*.* The number and category of single copy genes were equally distributed in both MD31 and MD32. Furthermore, two-way comparison using psi-Blast of ORFs among these three genomes and reference genome of *Sulfurospirillum deleyianum* DSM 6946 (NC_013512.1) shows that *Sulfurospirillum* MD31 and MD32 in comparison to *Sulfurospirillum* MD10 and S. *deleyianum* DSM 6946 have similar abundance and distribution of their sequence homolog (i.e. ORFs having >60% in amino acids; Figure S6). The close similarity between MD10 and MD31 is supported by 100% similarity of the partial 16S rRNA gene, while comparison of Average Nucleotide Identity (ANI) between MD10 and MD31 showed that the two genomes displayed 99% ANI (9,110 fragments in 200 bp step size and 1,000 bp reading windows), while MD10 and MD32 had 81.4% AVI (4,200 fragments).

**Autotrophic pathway “completeness”**

Certain key genes must be present in binned genomes for canonical autotrophic pathways to be considered “complete.” For example, according to RAST, two enzymes, RuBisCO and phosphoribulokinase, when present together, can confidently be used as indicators of the presence of the Calvin Cycle in a given organism. For the Wood-Ljungdahl (Reductive Acetyl-CoA) Pathway, the bifunctional carbon monoxide dehydrogenase/acetyl-CoA synthase enzyme (*acs*AB), which catalyzes the reactions from CO_2_ to CO and from CO_2_ to a methyl group, must be present for the pathway to be considered complete. Lastly, for the reductive TCA Cycle, four molecules of CO_2_ are fixed by three crucial oxygen-sensitive enzymes, all of which must be present for the pathway to function: ATP-citrate lyase, 2-oxoglutarate oxidoreductase, and pyruvate:ferredoxin oxidoreductase.

**Archived false positive (AFP)**

The following criteria were used for OTU retention based on AFP sequence distribution: 1) Any OTU whose maximum relative abundance in a sample was at least ten times higher than its abundance in the AFP was retained. The factor of 10 cut off was implemented based on the assumption that abundances below this threshold may represent cross-contamination during the failed PCR run that originated in the AFP, and 2) Any OTUs that shared the same top Blastn hit as a removed OTU, and whose maximum percentage abundance in a sample was less than its abundance in the AFP, were also discarded. This second step targeted OTUs generated from sequencing errors that might represent false diversity from laboratory contaminants. The dominant taxa associated with the sequenced AFP sample control are presented in Table S5.
